# Family in the experience of multiprofessional team care for elderly residents of a Therapeutic Residential Service

**DOI:** 10.1590/1980-220X-REEUSP-2025-0254en

**Published:** 2026-02-13

**Authors:** Aldair Weber, Giulia Delfini, Joaquim Manuel de Oliveira Lopes, Vanessa Pellegrino Toledo

**Affiliations:** 1 Universidade Estadual de Campinas, Faculdade de Enfermagem, Campinas, SP, Brazil.; 2 Universidade de Lisboa, Escola Superior de Enfermagem, Centro de Investigação, Inovação e Desenvolvimento em Enfermagem de Lisboa, Lisboa, Portugal.; 3 Universidade de Lisboa, Escola Superior de Enfermagem, Lisboa, Portugal.

**Keywords:** Aged, Patient Care Team, Family, Psychiatric Rehabilitation, Mental Health Services

## Abstract

**Objective:**

To understand the implications of the family in the experience of care provided by the multiprofessional team to elderly residents of Therapeutic Residential Services.

**Method:**

Qualitative research based on social phenomenology. Phenomenological interviews were conducted with 15 professionals from multiprofessional teams of Therapeutic Residential Services in a municipality in São Paulo state.

**Results:**

Experiences in caring for the elderly in Therapeutic Residential Services and their implications for families characterized the “reasons why” in the category “The family in the experience of multiprofessional teams caring for elderly residents of Therapeutic Residential Services.” These deinstitutionalization devices value living, where residents can strengthen family ties and establish new relationships. Future motivation is described in the category “Intentions of the multiprofessional team towards the family in the care experience.”

**Conclusion:**

Deinstitutionalization enables elderly residents to rebuild emotional bonds, with the team assuming the role of emotional reference in the biographical journey of their life world. The expectation of articulating care with the presence of family members aims to improve the experiences of elderly residents.

## INTRODUCTION

Psychiatric deinstitutionalization, pioneered in Italy, is considered an important milestone in the modernization of mental health care^(1)^. Based on this, in Brazil, the Brazilian Psychiatric Reform (RPB in the Portuguese acronym) drove the health reform that gave rise to the Unified Health System (SUS), transforming mental health policy in the country^(2,3)^. Law No. 10,216/2001, a reference in the anti-asylum struggle and the RPB, guarantees the rights of people suffering from mental illness and redirects the care model, which became territorial and community-based, structured by the Psychosocial Care Network (RAPS in the Portuguese acronym), whose purpose is to create, expand, and coordinate health care points within the SUS^(3,4,5)^.

The deinstitutionalization movement has promoted important changes in health care in several countries, allowing people suffering from mental illness to experience the aging process^(6,7)^. In this sense, care for the elderly is complex, with unfavorable socioeconomic conditions predisposing older adults to vulnerability, with adverse health outcomes and an impact on quality of life^(8)^. The presence of greater functional deficits, lower family income, and greater dependence on public services characterize the programmatic vulnerability of the elderly, a dimension that should be considered an important aspect in the planning of health service actions by reinforcing the need for networked care through coordination between different points of care^(8)^.

Due to the greater need of older adults for emotional support, financial assistance, and assistance with activities of daily living, family members often become caregivers^(6)^. When considering the care of older adults with severe mental disorders, it is possible to list some problems involving the family: the breakdown of family ties; the family as the basis for emotional development while also being the scene of conflicts, which can cause or intensify psychological suffering; and the role of caregiver, often associated with higher levels of stress and overload due to the high demand for care and lower rates of mental health, which directly impacts the quality of life and well-being of family members^(4,6,9)^.

In Western European countries, family dynamics tend to exclude the elderly, which reinforces the phenomenon of transinstitutionalization, characterized by the transfer of care from one institution to another - generally from psychiatric hospitals to nursing homes, which reduces the opportunities for these elderly people to benefit from a community-based model of mental health care, which enables them to acquire greater autonomy, improve social skills, and reduce psychiatric symptoms^(7)^.

In Brazil, in order to overcome the asylum logic and promote psychosocial rehabilitation, deinstitutionalization strategies were created, such as “De Volta para Casa” (Back Home), a program for the resocialization of people discharged from hospitalization^(6)^, and Residential Therapeutic Services (SRT in the Portuguese acronym), houses or dwellings in the community, included in the RAPS and aimed at people discharged from psychiatric and custodial hospitals, people with mental disorders who need assistance to maintain housing, who are homeless and/or who have lost social and family ties^(2,10,11,12)^. However, the vulnerability of older adults can be aggravated in the context of SRTs because, in isolation, these devices are unable to respond to the multiple demands of this population, which highlights the need for coordination with RAPS services and intersectoral actions to ensure comprehensive care^(3,8,13,14)^.

In order to accommodate the heterogeneity of residents, SRTs are divided into two types: I, for people with a lower degree of dependency, which can accommodate up to eight residents; and II, for those with greater care needs, accommodating ten residents^(10,11)^. It is suggested that each Therapeutic Residential Service (SRT) have at least five caregivers and one nursing technician for every ten residents^(11)^and be linked to a reference mental health service/team responsible for multiprofessional technical support^(11)^. The professionals’ role is to assist residents in reappropriating their living space, building daily living skills (self-care, nutrition, hygiene, among others), developing forms of communication, and improving conditions for establishing emotional bonds, in accordance with their respective individual therapeutic projects, with a focus on integration into the existing social network and in line with the psychosocial rehabilitation policy^(2,4,10,11)^.

One possibility for the multiprofessional team to act in this scenario is family-centered care, one of the pillars of the community model of mental health care in the context of deinstitutionalization, which benefits people with mental suffering by improving their overall functioning, social functioning, and communication, increasing adherence to treatment, and improving quality of life, in addition to being a source of security and support, which, as a consequence, can reduce the positive and negative symptoms of mental disorders^(15)^.

Deinstitutionalization strategies such as SRTs symbolize the change that has taken place in the Brazilian care model^(4,12)^. These RAPS care points represent a new possibility of living in freedom, welcoming vulnerable people who have been discharged from long hospitalizations and have fragile family ties^(2,5,11)^. In everyday life, mental health care permeates the nuances of the family, from the benefits provided to the elderly person suffering from mental distress to the overload experienced by family members as caregivers^(6,9,15)^. Thus, given the phenomenon of deinstitutionalization, the complexity of caring for the elderly, and the importance of SRTs, there is a knowledge gap regarding the singularities of family relationships in the care provided by the multiprofessional team in these deinstitutionalization devices in the community model of mental health care^(7,13,14)^, especially in the face of a backward movement due to the current strengthening of asylum practices^(16)^.

Finally, this study is justified by the important role of SRTs in the context of the Brazilian Psychiatric Reform as territorially based facilities for psychiatric hospital graduates, whose main purpose is to provide housing and develop care from a psychosocial rehabilitation perspective^(2,4,10,11)^, and by the problems faced by professionals in the multiprofessional team when considering the influence of the family context of elderly residents, often associated with the development of psychological distress, marked by broken bonds, stigma, exclusion, and illness among family members^(1,2,4,6,9,13,14,15)^. Therefore, the objective is to understand the implications of the family in the experience of care provided by the multiprofessional team to elderly residents of SRTs.

## METHOD

### Study Design

This is a qualitative study based on the theoretical-methodological approach of Alfred Schutz’s phenomenology, which aims to understand the phenomenon from social action, considered as an intentional act shaped by individual motivations within the world-life^(17)^. This is understood as an intersubjective scenario inhabited by humans, in which they develop their actions and interactions and give them meaning, influenced by subjective face-to-face interactions and a biographical trajectory, which together make up the body of knowledge^(17)^. Schutz proposes understanding the motives of previous experiences (“reasons-why”) and future expectations (“reasons-for”) of people in the intersubjective scenario they inhabit and who establish interactions through face-to-face relationships, constituting their life-world^(17)^. Based on motivations, the lived type of the person in the social world is constructed, and common patterns of meaning are conceived that aid in the understanding and interpretation of lived reality^(17)^. The recommendations of the*Consolidated criteria for reporting qualitative research - COREQ* ^(18)^were followed.

### Location

The study was conducted in a municipality in the state of São Paulo, which has a Psychosocial Care Network (RAPS) composed of services such as psychiatric beds in a general hospital, Psychosocial Care Center (CAPS) III, CAPS Alcohol and Drugs, CAPS Children and Adolescents, SRTs, among others^(19)^. The SRTs are linked to the CAPS III in the territory where they are located and are organized as follows: in terms of staff, type II centers have mid-level professionals (health caregivers or monitors) available 24 hours a day and nursing technicians, while type I centers have a mid-level worker working only during one period of the day^(11)^. Three CAPS III in the municipality agreed to participate in the data collection, two located in the Southwest health district and one in the Northwest^(19)^. They are responsible for monitoring five type I and two type II SRTs, housing around 18 residents in total.

### Participants

This study involved professionals from the multiprofessional team working at CAPS III who provide direct care to the elderly in SRTs, including nurses, psychologists, occupational therapists, social workers, nursing technicians, monitors, and housing assistants. Snowball sampling was used, in which one participant refers another to the professionals^(20)^. The three managers of each CAPS III, referred to as seeds, referred the first participants they considered to have experiences related to the study theme, thus favoring the construction of a network of trust and credibility throughout the process^(20)^. Prior contact, invitations to participate, and scheduling of interviews were done both in person, during visits by the researcher to the service, and via electronic message through a mobile application, with the support of the managers.

The following inclusion criteria were considered: belonging to the multiprofessional team that provides care to elderly residents of the SRTs, having at least six months of experience, and not being on vacation or away from work during the data collection period. Professionals who do not provide direct care to residents, such as pharmacists, pharmacy technicians, hygiene staff, security service, and administrative assistants, did not participate in the study.

### Data Collection

Data collection was carried out from January to December 2024 through phenomenological interviews conducted by the first author, a doctoral student in health sciences and nurse at the time, with extensive academic experience in the methodology employed. These interviews allow participants to report to the interviewer the meaning attributed to the phenomenon experienced and the action performed in their context of social interactions^(21)^. The trigger questions were: “Have you ever cared for an elderly resident in the SRT?”, “Tell me how it was and what the implications of the family were in this care,” and “What was your intention in providing this care?” Based on these questions, other questions were developed that allowed participants to delve deeper into the topic and share their experiences. No pre-testing of the trigger questions was conducted, as they were defined with the support of the research team before data collection began. At the end of the interview, information was collected on the length of time the participants had been working in the SRTs and their work experiences.

Only the researcher and the interviewee were present during the interviews, which took place in various locations, such as the CAPS III premises, the SRTs, and external spaces. They were conducted by the principal investigator and recorded on audio, with an average duration of 25 minutes. There was one withdrawal, justified for personal reasons, at which point the researcher turned to the seed^(20)^, obtaining a new participant referral. No repeat interviews were conducted. Data collection was concluded when the principal investigator understood that the phenomenon had been revealed, the concern had been resolved, and the ideas in the statements began to repeat themselves, thus providing sufficient subjective meaning structures to describe the typical action. At that point, no new significant elements emerged in the data, such as nuances, dimensions, or variability^(17,22,23)^.

### Data Analysis and Treatment

In order to ensure the rigor of the data analysis process and understanding phenomenology as a continuous movement, at all stages we returned to Schutz’s main concepts (social action, world-life, biographical trajectory, knowledge base, face-to-face relationship, reasons-why and reasons-for)^(17,23)^. This constant return is justified because the process is mediated by the researcher’s biography, requiring the mental exercise of distancing oneself in order to adopt a scientific attitude. When interpreting and systematizing the subjects’ statements, the lived type is elaborated, that is, the action of objectifying the interviewees’ subjective views^(23)^. This elaboration is made possible through*epoché*- suspension of the researcher’s assumptions and focusing attention on their concerns and the objectives of the study - and eidetic reduction, which consists of conscious analysis based on the units of meaning of each individual^(23)^. Thus, in phenomenology, knowledge is constructed from what is experienced in common sense, and it is up to the researcher to organize the subjective data, describe the experience, and bring it into the order of meanings^(23)^.

**Figure 1:**
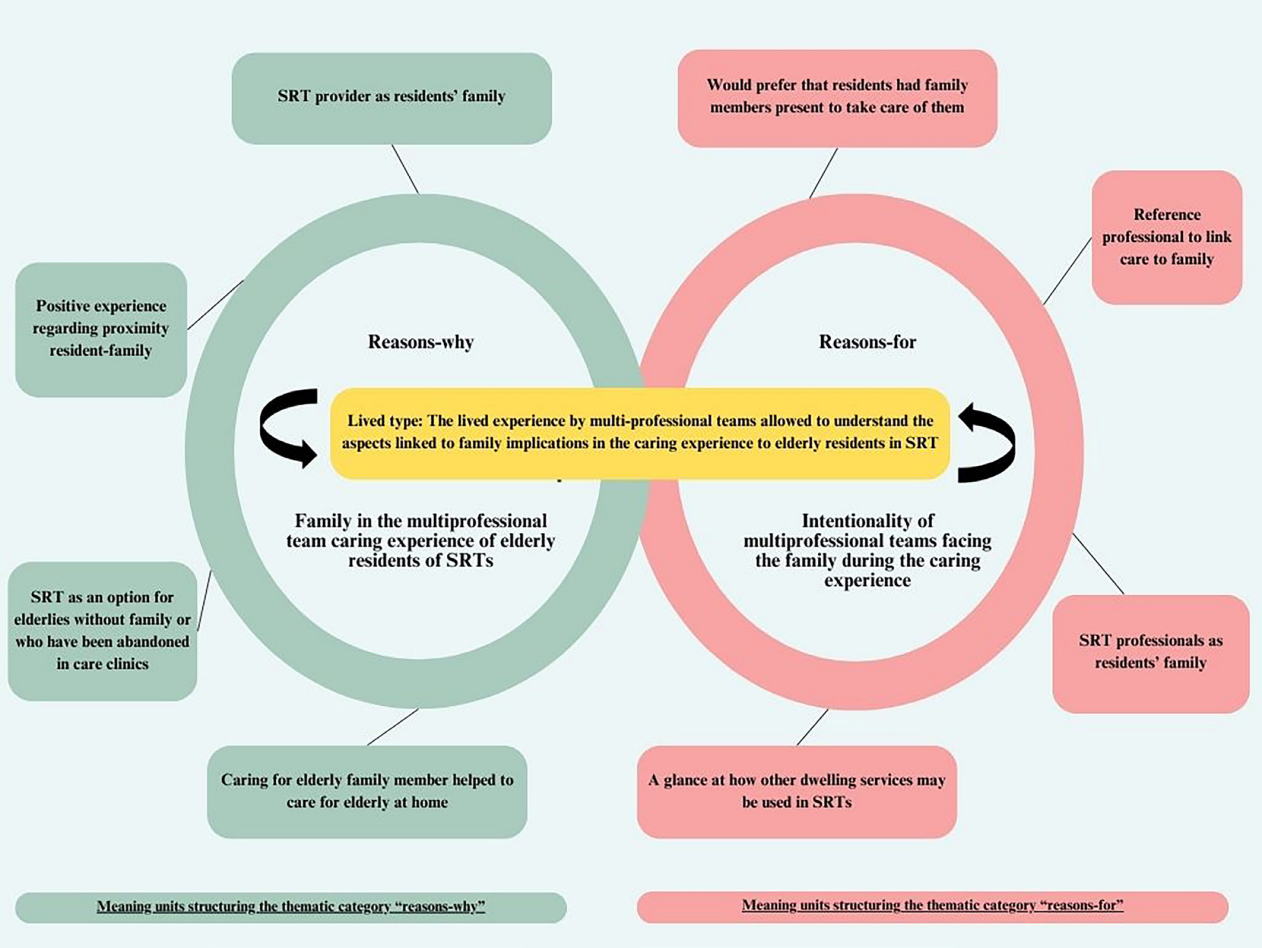
Summary diagram of the data analysis process.

Based on the theoretical framework of social phenomenology^(17)^, the data from the interviews were analyzed by the principal investigator and validated by the research group to which he is affiliated, without the use of*software*, following the steps recommended in studies based on phenomenological analysis and Schutz’s concepts^(17,23)^: full transcription of each interview, organizing the text by subject based on the guiding question, with the aim of learning the individual meaning; continuous reading and rereading of the transcripts to facilitate a global understanding of the participants’ experiences; excerpts from the statements that highlight the structures of the subjective meanings of the action, gathering the excerpts that reveal similar motives; formulation of categories of human action in relation to the meaning of the phenomenon by grouping excerpts from the statements, distinguishing them into “reasons-why” - explaining the performance of a certain action, and “reasons-for” - corresponding to the intentions or purposes that guide the action, as illustrated in Figure 1.

### Ethical Considerations

This study was cleared by the Research Ethics Committee of the State University of Campinas (UNICAMP), under Opinion No. 4.501.808. Participants were informed that this was a scientific investigation whose theme was experienced by the principal investigator in his daily life as a nurse, thus seeking new reflections on this topic to qualify professional practice. In accordance with Resolution No. 466/12, participants signed the Free and Informed Consent Form and the Voice Recording Authorization Form and were informed that this was an academic research study. In order to preserve anonymity, the reports were identified with the letter “E” for “interviewee,” followed by the number corresponding to the sequence in which the interviews were conducted.

## RESULTS

Fifteen participants took part in the study, including four nursing technicians, four monitors, two nurses, two occupational therapists, one psychologist, one social worker, and one housing assistant. The average length of service in the SRTs was five years. In terms of work experience, in addition to working in mental health, the participants were also or still were: caregivers for the elderly in home care and nursing homes; linked to other health institutions; or professionals in sectors not directly associated with health, such as domestic workers, customer service, and education.

Based on the interviews, it was possible to organize the results into two categories that constitute the participants’ lived experience in the social world through the motivations described below: “The family in the experience of the multiprofessional team caring for elderly residents of Therapeutic Residential Services,” which characterizes the “reasons-why” of the intersubjective meaning of the participants’ experiences in the world and the family implications of caring for the elderly in the SRT from the perspective of professionals; and “Intentions of the multiprofessional team towards the family in the care experience,” to describe the “reasons-for” the care provided by the team as social action, their intentions, and what they expect from their actions based on face-to-face encounters.

### The Family in the Care Experience of the Multiprofessional Team for Elderly Residents of Therapeutic Residential Services

The multiprofessional team identifies that elderly residents of SRTs often do not have family support for care provision and that there are few cases in which it would be possible to reconnect these residents with their families, who sometimes choose to refer the elderly to nursing homes. They refer to these aspects as abandonment and rejection, reinforced by the difficulty of establishing contact with family members, who distance themselves from care because they are unable to cope with a dependent individual and due to histories of family conflict.

*Most of them do not have family support. [...] When they are in the residence, they will have all the support that they do not have at home. [...] Of the residents we have today who have contact with their families [...] three or four, and I’m talking about a very high number, would be able to return to their families. – (E6)*

*Most of them don’t have families, and if they do, over time, they no longer provide care, they’re not there. So, I think this project is great, and a therapeutic residence, you know? Most of them, for some reason, or abandonment [...] the family ends up putting them in a clinic, because they say they can’t take care of them, because before they could walk, they were independent, now we can’t take care of them, so we’ll put them in a clinic. [...] In the case of the therapeutic residence, these are patients who don’t have families. – (E11)*

*Today, with this family estrangement, I’ve tried several times to get closer to my daughter, and she doesn’t even respond, she doesn’t answer my calls, she doesn’t want to know about her mother. [...] Because often there’s also this story of the elderly person with their family, we don’t know how it was for this elderly person. There’s the issue of abandonment, rejection. And when they reach this age, these people who have been through all this need to go through all these feelings and deal with them. Many can’t, so they don’t want contact. [...] Most elderly people have to deal with this issue of abandonment, and I realize that she tries to stay away because reality hurts too much. – (E15)*

In the context of deinstitutionalization proposed by the RPB, the support and social and family ties of those leaving psychiatric wards are often worn thin, and the SRT emerges as an alternative form of housing so that these people do not live on the streets, subject to violence and vulnerability. The service is seen as a potential that values living, where residents are assisted and supported. However, for the participants, the SRT should only be considered when investments in the family nucleus have been exhausted.

*With this whole reform project, the closure of beds and deinstitutionalization, we thought collectively and created the therapeutic residence project for patients discharged after long periods of hospitalization, who no longer had social ties, no longer had family ties, and who were no longer going to live in hospitals and would have to live somewhere. [...] Family units that are very worn out, where the relationship between the patient and the family network is very strained [...] refer them to be included in the therapeutic residence program, or this patient would end up homeless or subject to violence and social vulnerabilities, and their life situation would worsen. [...] We have to invest what we can in this*(family)*unit so that this patient can return to living well and living in this unit. If there is no possibility, after exhausting all investments, we consider therapeutic residence. – (E4)*

*They never had a home, they never had that support, that care, they feel like strangers. [...] I’m talking about someone over 60 years old who has no family support. They don’t know what it’s like to go for a walk in a square or a park because they never had that opportunity. I think this is a huge potential. I think we can offer it as CAPS, as housing, I as a reference for the home too, to value this living. – (E10)*

*Because they are residents who have no family, they are aging, and they are very well cared for, they are assisted, so I don’t think they are helpless. – (E8)*

In some cases, professionals report good experiences with elderly SRT residents who still maintain ties with their family nucleus, whose family members visit the housing, which contributes to the restoration of the person’s autonomy. When this is not the case, residents, as part of their psychosocial rehabilitation, seek other forms of family that are not blood-related, such as when the community understands and inserts itself into the context of these elderly people’s lives through donations and when they take care of and protect themselves.

*We have had good experiences with this process with patients who still have a family unit and whose family visits them at home, goes to their birthday parties, and participates in celebrations, but who live in a therapeutic residence and whose family lives in their own space. This is what enabled the patient to reestablish a more autonomous life process. – (E4)*

*I keep thinking about rehabilitation, which ranges from financial issues to family ties, which are often broken, but we are left with this more visceral family issue, and they create other families, other networks, which also add a lot, and I believe that this is part of rehabilitation. [...] The parties are 100% donations. I think it’s fantastic, because it’s the bakery, the restaurant that donates, the market, the bar, the families. The neighbor who works on the farm says, “I hear there’s going to be a party. Stop by and pick up two cartons of eggs.” So, that’s taking ownership. That’s us making the community look and understand. – (E15)*

*When they leave the gate, they take care of each other. [...] They hold hands, they help each other [...] Whereas inside the house they fight over anything, outside they don’t, outside they protect each other. I think that in their little heads, it’s my family. So, I’m going to take care of them, and they take care of each other very nicely. – (E2)*

Finally, the multiprofessional team, in addition to often considering themselves the family of the elderly residents of the SRT, because they bring the care they offer closer to personal experiences, also believe that the residents see them as family, because they put the team in this place, want to be close to them all the time, and show gratitude, respect, and affection for the professionals. As a result, while they suffer from the death of elderly residents, as they spend more time at work than at home, they also consider that residents would like to have someone who is actually family in times of frailty and illness, because they believe that it is not possible to fill that role as professionals.

*My mother was bedridden, and I already learned a little, so I came here already knowing how to care for an elderly person. – (E1)*

*Many have family, others have no one... Their family sees us as their family, right? So, when you get there, when you haven’t been there for a long time, the day you show up, they come and tell you things. [...] And you see that they want to be close, all the time. – (E3)*

*I think that of all the stops in the way, the places we call home, housing is the most important. Because in the environment, patients come, stay in the environment, and go home... Not in the residence. What they have as a family base, let’s say, is the staff, the monitors, the housing assistants, it’s us who go there to medicate them. I think we are their family. They don’t have a lot of stress. Although some still have contact with their family, someone far away, but I think that’s it. – (E6)*

*This makes me always walk in mental health, this gratitude that they never received. This family that they don’t have, we try to provide, but we need to have this view of the elderly that goes far beyond. – (E10)*

*They look at us with affection. They have a lot of respect for us. Some of them say things like... “Oh, I remember my mother. She used to take care of me like this, right?” [...] And I also find it very sad when they pass away. [...] It’s as if we had lost someone in our own family. Because we spend more time here than at home. [...] They see us as family. [...] Now, I don’t think they think that way. I wish that when things are bad... Their family, someone by their side, there’s no one there. That’s when it gets to us. [...] – (E7)*

*We also can’t fantasize and try to occupy other places in their history, which I think is also about that, I think mental health has that, that awareness, that reconnection, with life and with family and everything else, but it’s also part of their history. – (E5)*

### Intentions of the Multiprofessional Team Towards the Family in the Care Experience

Based on the idea of considering elderly residents as family members, the team intends to provide care in the best way possible, with affection and in the way they would like it to be done with them. In cases where they have a family network, it is important for the reference professional to coordinate care with the family, in addition to the other RAPS services that the resident receives.

*It’s good, I like it, and I try to care for them with the utmost affection, as if they were one of my own, part of my family. So I try to do my best, as I would like to be treated, I try to treat others. – (E12)*

*Discussions about housing need to take place within the CAPS. The referral needs to be made together with the nurse from the CAPS mini-team, together with the doctor. The referral needs to coordinate this care with the network. So, whether it is with UNICAMP, with Mário Gatti, with the health center, with the family, because some have family, others do not. And we can accompany them in these last moments of life. – (E14)*

The team would like the elderly residents to have family, so that they can stay together and have someone to visit, despite the difficulties of living with them. From this, they identify that the SRT professionals are the family of these residents.

*I would like them to have family, all of them. I think that, no matter how difficult it is to live with your family, there is nothing else like it, no other feeling like it. So, I think the first thing was for them to have someone there for them, you know? Family, so they could be together, go to their house, all of them. Go out, be able to go... I went to my mother’s, I went to my aunt’s, I went to someone’s... I went to someone’s house, a family member’s. – (E3)*

*The family doesn’t accompany them. We end up being the family for them. That’s what keeps me going. – (E13)*

One of the team members reported a unique experience during their training, when they learned about housing services in another country, a model in which houses are distributed by the municipality and the team goes to the residence based on the residents’ demand, envisioning this model for SRTs in Brazil.

*I had the experience of going to Portugal and learning about Housing First, for example, which is a different approach. We know that we are talking about another country, another culture, another public policy. So, we can’t just compare, copy, and paste the project here. [...] Maybe I can envision having that experience here. There, they are individual houses, or an individual or families have their own houses distributed throughout the municipality, and they did this through visits or through demand. [...] So, to clean the house, buy groceries, organize finances, sometimes, some follow-up with a health service. So, there were professionals who were references for the scattered houses. As a reference, I would have five houses to take care of. But I would have the week for that care. – (E9)*

## DISCUSSION

The approach to the experience of the professionals who make up the multiprofessional team allowed us to understand some aspects related to the implications of the family in the experience of caring for elderly residents of SRTs. The social actions of the participants revealed the motivations that exist in their world-life, in which relationships with elderly residents in the daily care of the SRT build the intersubjective space that allows them to aggregate knowledge and pave the biographical trajectory^(17)^.

One of the main findings of this study concerns the lack or fragility of family support for elderly residents of SRTs. Family composition is particularly important, given that the family is considered the most basic social unit in human life and part of people’s life world, closely related to the provision of physical, psychological, and spiritual support, in addition to offering daily care. Feelings of loneliness and abandonment are common among the elderly population. These are complex issues with profound implications for well-being and mental health, requiring integrated approaches and intersectoral coordination that consider individual, relational, and contextual aspects^(24)^.

It should be noted that older adults are vulnerable in individual, social, and programmatic contexts^(8)^. Those who have been discharged from long-term psychiatric hospitalization are more likely to lose family ties or have fragile relationships^(10)^. This reality, associated with the consequences of the asylum model, is one of the criteria for admission to SRTs^(11)^. Thus, the fragility or lack of family support among elderly residents of these facilities reveals a significant rupture in their life world. In this sense, living in an SRT does not only mean having care and housing, but represents a response by public policies to the programmatic vulnerability of the elderly^(8)^, in addition to enabling the reconstruction of intersubjective networks and the reframing of trajectories^(17)^.

Family care is part of the world-life, and people who receive it have a better prognosis for health and quality of life^(6)^. However, the burden and responsibility assigned to family members are also associated with high levels of psychological distress^(6)^. Caring for an elderly person with psychosis, for example, in whom organic comorbidities are common, challenges the available knowledge base, demands more, and is more stressful, since it breaks with the typification associated with typical aging^(6)^. It is pointed out that family members who care for elderly people suffering from psychological distress experience emotional exhaustion, difficulty in coping with behavioral symptoms, and identify the need to share the responsibility of care with health services in an attempt to alleviate the burden^(25)^. Therefore, the mental illness of caregivers due to care overload can even affect their ability to care, their quality of life, their self-esteem, and cause them to behave in a hostile manner, in a vicious cycle that can destabilize the relational fabric that sustains the social world, generate family conflicts, and negatively impact the health of the person being cared for^(6)^.

Although it is the family’s duty to support the elderly, family members do not always have the knowledge necessary to provide the care required during the aging process, with its physical and psychosocial changes in the world-life^(26)^. This situation is intensified in today’s society, where families are becoming smaller, the pace of work is more intense, and care almost always falls to one family member^(26)^. In this context, by straining the traditional typification of the family role, it is common to delegate care to long-term care institutions, for example, with these new intersubjective networks often considered by family members as a more suitable environment to meet the health needs of the elderly^(26)^. However, elderly people with severe psychological distress are highly vulnerable and have specific needs for long-term care. They often face significant barriers to admission to long-term care institutions, which generally have difficulties in offering specialized mental health support, compromising the quality of care^(27)^and reinforces the phenomenon of transinstitutionalization^(7)^and highlights an important challenge that has not yet been overcome^(27)^.

For elderly people with mental suffering who have fragile family and social ties, the SRT presents itself as an alternative space that values living, providing an intersubjective environment for the development of social and emotional relationships for those who were previously confined to psychiatric hospitals, with the main objective of restoring autonomy and the right to live in the community through psychosocial rehabilitation. Thus, people who were previously considered patients are now residents^(2,28)^in the SRT and, based on their knowledge, can rebuild new paths in their life world^(17)^. Care in an asylum regime was marked by limitations of space and individuality, which were transformed for SRT residents into opportunities to circulate in the community, live and interact with other people through face-to-face relationships, exchange experiences, and, above all, learn from each other and the social world around them^(17,28)^. In this process, the action of the multiprofessional team is fundamental to stimulate living in the territory of the SRT residents, the exercise of citizenship, and the configuration of this space as a home and not a place of treatment^(29)^.

In addition, the feeling of belonging and inhabiting a space is of great significance to people suffering from mental illness, since living is constituted in the world-life as an intersubjective construction loaded with meaning^(17)^and “home” is commonly a place of Welcoming, comfort, intimacy, exchanges, and experiences of affective and interpersonal relationships based on accumulated knowledge, legitimized through daily domestic life among the elderly residents of the SRT^(28)^. The living space must adapt to the intentions of its residents and provide flexible support from professionals, beyond external standards of organization, since “being at home” represents an opportunity to express their subjectivities and exercise their freedom^(13)^. From this, a new understanding of “living” is constructed: now a subjective construction based on the biography of each resident, who has a place to call “their own” in the world-life^(2,13,28)^.

In some cases, bonds between elderly residents and their family members, who visit the SRT, are still identified. The asylum model produces exclusion and distancing from the social world, with an impact on family relationships from the first psychiatric admissions, as these people usually do not receive visits while they remain in institutions, which harms social and emotional relationships^(13)^. Frequent interaction between family and the elderly through visits to the SRT, in cases where they do not live together, does not meet the resident’s interpersonal communication needs. However, this closeness still promotes physical and mental health, updates knowledge, and reinforces the feeling of belonging and biographical continuity in the world-life, in addition to enabling a more positive perception of the aging process, which impacts quality of life^(24)^. As pointed out by the participants, such visits are characterized as face-to-face encounters and represent positive impacts as opposed to the typical exclusion of asylum practices, which can help motivate future actions by residents and the multiprofessional team^(17)^.

In order to fill the gap of fragile or non-existent family relationships, elderly residents seek other forms of family, whether inside or outside the SRT, in a movement to occupy the territory, rebuild belonging in the world-life, and update their stock of knowledge^(17)^. By living in the same residence, the elderly develop a sense of belonging and of living in a collective, reframing family typification and expanding affective, intersubjective, and social relationships^(17,28)^. In the context of psychosocial rehabilitation, the act of living also becomes a form of material and symbolic appropriation of the spaces of the home, where coexistence is consolidated as a process of exchange of resources, care, and affection among residents^(28)^. However, there is a centrality of SRTs in the health services network, which can restrict the social reintegration of residents and hinder the creation of new bonds^(13)^. This fact points to the need to think about care actions from an intersectoral perspective^(3,8,13)^.

In addition, overcoming the asylum model requires the involvement of elderly residents in the city, given the porosity of the urban fabric provided by SRTs, through insertion into social groups and/or public and community spaces, which promotes the expansion of the world-life and the reframing of social typification associated with exclusion^(17,28)^. This form of social participation strengthens intersubjectivity and can transform the daily life, biographical trajectory, and living conditions of the elderly, which are often marked by illness, violence, social injustice, inequality, prejudice, exclusion, and oppression^(28)^. In social reintegration, community attitudes are fundamental, especially when seeking to promote mental health awareness through direct contact with SRT residents, actions that help reduce stigma and strengthen the model of care in freedom^(30)^. As a consequence of living in society, residents expand their interpersonal communication, cultivate hobbies, and improve their cultural quality by valuing the subjectivity and potential of encounters.

In this context, the professionals themselves consider themselves family to the elderly residents. Autonomy in SRTs, when compared to nursing homes, is superior mainly due to the construction of interdependence between devices, territory, and people, with the latter also considering the face-to-face relationship between residents and professionals to produce new meanings^(28)^. The construction of these relationships is an integral part of the deinstitutionalization process, since the possibility of making choices allows for biographical reconstruction and the reframing of meanings and knowledge stores of their life world^(17,28)^. In addition, the actions of professionals in everyday life go far beyond physical and psychosocial issues. It is a perspective of relational and therapeutic care, expressed through qualified listening, interpersonal communication, and the construction of bonds with new meanings in the life world, which also provide emotional and spiritual comfort^(17,31)^. This interaction reveals the potential of the multiprofessional approach to mental health through person-centered care and the valorization of subjectivity^(31)^, which are fundamental aspects for psychosocial rehabilitation.

However, professionals must be careful when considering elderly residents as family members, as this demonstrates ambiguity in their social and therapeutic functions, which are necessary for their work in the SRT^(2,10)^. This attitude of caring is associated with charity, restricts intersubjectivity, and reproduces the idea of social isolation historically perpetuated in mental hospitals, since it is necessary to reframe care in order to consider that these devices require intersectorality and technical knowledge, aligning them with motivations oriented towards strengthening and building the world-life of elderly residents^(2,10,17)^. Although they exist, intersectoral actions in the context of care in the RAPS occur through shared care and meetings, often in a restricted and isolated manner, without effective systematization^(3)^. This context reflects a fragmented network centered on specialized services, but which needs stronger links between levels and services to ensure longitudinal, territorial, and technically based care^(3)^. Thus, phenomenological understanding contributes to revealing that the care provided by the multiprofessional team is permeated by a charitable attitude, a characteristic associated with people’s previous experiences and with strong social appeal, which may distance itself from practices based on technical and scientific knowledge^(17)^.

Thus, it is possible to highlight that the current model of mental health care still faces obstacles in the implementation of care in freedom in the territory^(3,13,29)^. Proposals that encourage psychiatric hospitalization and the financing of therapeutic communities based on a prohibitionist and exclusionary view of people suffering from mental illness still persist^(16)^. Such actions reflect attempts to resume the asylum model, which reinforces the importance of critical reflections on practices in health services^(13,16)^. In this context, the action of professionals stands out, whose performance must be aligned with the principles of psychosocial rehabilitation and the guarantee of rights, in order to overcome institutional logics that are still in force^(13)^.

Professionals report providing care to elderly SRT residents in the way they would like it to be provided to them, which demonstrates empathy. This is a multidimensional construct that includes cognitive and affective factors and is considered to be the professional’s understanding of the resident’s feelings, an essential aspect in mental health contexts, in which people in psychological distress value the professionals’ perspective and their understanding of the situation^(32)^. The empathy exercised by SRT professionals emerges from the shared world-life and face-to-face relationships, allowing them to understand the residents’ experiences based on their knowledge and typification of what they have experienced^(17)^. Empathetic capacity also allows for the adoption of non-defensive positions and facilitates the achievement of satisfactory and productive results^(32)^. However, in the context of mental health care, empathy cannot be seen only as a personal characteristic, but as a relational competence that becomes a central element of the therapeutic relationship^(31)^, enabling qualified listening, recognition of uniqueness, and strengthening of bonds with SRT residents. Thus, empathy can be understood as a perspective that should permeate the work of the entire multiprofessional team, as an essential aspect of care^(32)^.

Participants identify the importance of coordinating care with family members and other services that the elderly resident uses, which represents a process of expanding intersubjectivity and updating knowledge about the uniqueness of residents, and demonstrates the need for SRT professionals to provide guidance, education, and support to family members and workers in other health contexts^(33)^. This practice can be understood as a strategy to address the programmatic vulnerability of older adults and the fragmentation of the service network, by enabling professionals to coordinate actions to expand access, strengthen bonds, and ensure continuity of health care^(3,8)^. As a result, there may be a rapprochement between the resident and the family context and other spaces in the community, in order to contribute to social participation and the reorganization of the world-life of the elderly, in line with projects aimed at autonomy and inclusion^(28)^.

Other comments from professionals were that they would like elderly residents to have family and, therefore, they put themselves in the place of family members. Empathy is an important factor in the development of the relationship between residents and professionals, as it strengthens face-to-face relationships and trust in care, and caution must be exercised not to exceed the limits of typical social roles, which are fundamental to the clarity of functions in the SRT^(2,10,32)^. As an alternative, in line with psychosocial rehabilitation, professionals can use residents’ knowledge to help rescue, recognize, and validate the elderly’s desire to reconnect with friends, family, places, and relationships they experienced throughout their lives before psychiatric hospitalization, with the aim of reviving moments of happiness and protection^(28)^.

Finally, professionals identify the desire for SRT care to be provided outside institutions, for example, through visits to homes scattered throughout the territory, which reveals actions oriented toward future motivation that aim to build practices aligned with the resident’s world-life^(17)^. This idea is in line with initiatives around the world, such as Housing First, which, unlike the transinstitutionalization movement evident internationally^(7)^, prioritizes immediate and independent housing, values autonomy and personal choice^(34)^, characteristics that increasingly aim at care outside hospitals and community institutions, carried out in the person’s own home, when possible^(35)^. To this end, health professionals need to become active agents in discussions about social isolation and mental health needs, especially among the elderly population^(35)^. It is revealed that the future motivations of the participants lie in the intention to care for mental health in freedom at different stages of life by valuing the biographical trajectory, shedding light on issues such as the isolation of elderly people in psychological distress^(17)^.

This study highlights the practices of the multiprofessional team in the context of the implications of the family in the daily care of elderly residents of SRTs, emphasizing the importance of this dimension in their life world. The approach focused on the singularities of the elderly public in psychosocial rehabilitation devices points to the relevance of broadening the discussion on the intersubjective needs of these people and qualifying professionals for care, including the nursing team, in view of the scenario of population aging.

Regarding the limitations of the study, it should be noted that the participants are professionals who work in some SRTs in the municipality where the research was conducted, and the results are linked to the experiences, perceptions, and specific contexts of the subjects involved^(17)^. However, this study did not set out to analyze other characteristics that may influence the life world and daily activities in SRTs. It is recommended that future research explore other structural, historical, and social aspects, especially in relation to the participation and role of the family in the process of deinstitutionalization and psychosocial rehabilitation of older adults, broadening the understanding of the challenges and potential of this care in different sociocultural realities.

## CONCLUSION

In this study, it was possible to construct the lived type based on the thematic categories that highlighted the motivations of the participants’ experiences, which were permeated by the implications of the family in the life world of the elderly SRT resident, which influences the care provided by the multiprofessional team.

Based on the results, it is noted that the biographical journey of the elderly residents of the SRT is marked by vulnerability, absence, fragility, and the breakdown of family ties, placing the SRTS as important spaces in the process of deinstitutionalization and psychosocial rehabilitation. The daily work of professionals through qualified listening and empathy makes it possible to reframe the residents’ experiences of exclusion and suffering. In this context, it is observed that the breakdown of relationships in the world-life makes SRTs a powerful alternative for housing and care, with the possibility of welcoming and rebuilding affective, intersubjective, and social bonds, either between residents who still maintain contact with their families of origin or through the formation of new relational arrangements within the home itself or in the community. The multiprofessional team also recognizes its role as an emotional reference and coordinator of actions in the residents’ world-life to ensure qualified and humanized care based on their past history and the reconstruction of meaning in everyday life. Despite this, in moments of greater emotional fragility, both the team and the residents would like to have the support of family members.

The intentionality of the actions of the multiprofessional team in the SRTs is marked by the search for qualified care for elderly residents. When family members are present, even if permeated by conflicts, the team expects the reference professional to coordinate the relationship between them, in addition to the expectation that residents can count on their own families. However, the different ways in which these spaces are organized also influence experiences, indicating that the way the service is structured can enhance or limit the relationship with the family.

In addition, the findings of this study point to the need for multiprofessional care in SRTs, especially by professionals who are closest to the residents, such as the nursing team, to be guided by expanded action and intersectoral coordination, recognizing the uniqueness of residents with a view to overcoming fragmented practices in the network’s services. Thus, these actions can be understood as fundamental strategies in addressing the programmatic vulnerability of older adults. Finally, the yearning expressed by professionals for territorialized and integrated care models reveals the motivation for action aligned with the principles of psychosocial rehabilitation, the exercise of citizenship, and the right to exist in society.

## DATA AVAILABILITY

The entire dataset supporting the results of this study is available upon request to the corresponding author.
