# Supplementary material for: Family in the experience of multiprofessional team care for elderly residents of a Therapeutic Residential Service
Source: Rev Esc Enferm USP. 2026 Feb 13;60:e20250254. doi: 10.1590/1980-220X-REEUSP-2025-0254en (PMC12904634; doi:10.1590/1980-220X-REEUSP-2025-0254en)
Supplement: Translation

# Translation: Família na experiência de cuidado da equipe multiprofissional ao idoso morador de Serviço Residencial Terapêutico

**DOI:** 10.1590/1980-220X-REEUSP-2025-0254pt

**Authors:** Aldair Weber, Giulia Delfini, Joaquim Manuel de Oliveira Lopes, Vanessa Pellegrino Toledo

**Affiliations:** 1 Universidade Estadual de Campinas, Faculdade de Enfermagem, Campinas, SP, Brasil.; 2 Universidade de Lisboa, Escola Superior de Enfermagem, Centro de Investigação, Inovação e Desenvolvimento em Enfermagem de Lisboa, Lisboa, Portugal.; 3 Universidade de Lisboa, Escola Superior de Enfermagem, Lisboa, Portugal.

**Keywords:** Idoso, Equipe de Assistência ao Paciente, Família, Reabilitação Psiquiátrica, Serviços de Saúde Mental

## Abstract

**Objetivo:**

Compreender as implicações da família na experiência de cuidado da equipe multiprofissional ao idoso morador de Serviços Residenciais Terapêuticos.

**Método:**

Pesquisa qualitativa fundamentada na fenomenologia social. Foram realizadas entrevistas fenomenológicas com 15 profissionais de equipes multiprofissionais de Serviços Residenciais Terapêuticos de um município do interior paulista.

**Resultados:**

As vivências no cuidado ao idoso no Serviço Residencial Terapêutico e suas implicações familiares caracterizaram os “motivos-porque” na categoria “A família na experiência de cuidado da equipe multiprofissional ao idoso morador de Serviços Residenciais Terapêuticos”. Esses dispositivos de desinstitucionalização prezam pelo morar, onde os moradores podem fortalecer laços familiares e estabelecer novas relações. A motivação futura encontra-se descrita na categoria “Intencionalidades da equipe multiprofissional diante da família na experiência de cuidado”.

**Coclusão:**

A desinstitucionalização possibilita ao morador idoso reconstruir vínculos afetivos, com a equipe assumindo o papel de referência afetiva no percurso biográfico do seu mundo-vida. A expectativa de articular o cuidado com a presença dos familiares visa qualificar as vivências dos moradores idosos.

## INTRODUÇÃO

A desinstitucionalização psiquiátrica, pioneira na Itália, é considerada um importante marco na modernização do cuidado em saúde mental^(1)^. A partir dela, no Brasil, a Reforma Psiquiátrica Brasileira (RPB) impulsionou a reforma sanitária que deu origem ao Sistema Único de Saúde (SUS), transformando a política de saúde mental no país^(2,3)^. A Lei nº 10.216/2001, referência da luta antimanicomial e da RPB, assegura os direitos da pessoa em sofrimento psíquico e redireciona o modelo de cuidado, que passou a ser de base territorial e comunitária, estruturado pela Rede de Atenção Psicossocial (RAPS), cuja finalidade é criar, ampliar e articular pontos de atenção à saúde no âmbito do SUS^(3-5)^.

O movimento de desinstitucionalização promoveu importantes transformações no cuidado em saúde em diversos países, permitindo que pessoas em sofrimento psíquico vivenciem o processo de envelhecimento^(6,7)^. Nesse sentido, o cuidado à velhice mostra-se complexo, em que condições socioeconômicas desfavoráveis predispõem a pessoa idosa à vulnerabilidade, com desfechos adversos na saúde e com impacto na qualidade de vida^(8)^. A presença de maior déficit funcional, menor renda familiar e maior dependência dos serviços públicos caracterizam a vulnerabilidade programática da pessoa idosa, dimensão que deve ser considerada como um aspecto importante no planejamento das ações dos serviços de saúde ao reforçar a necessidade de cuidado em rede por meio da articulação entre diferentes pontos de atenção^(8)^.

Devido à maior necessidade da pessoa idosa para apoio emocional, assistência financeira e desempenho de atividades de vida diária, os familiares, muitas vezes, se tornam cuidadores^(6)^. Ao considerar o cuidado ao idoso com transtornos mentais graves, é possível elencar algumas problemáticas envolvendo a família: a quebra de vínculos familiares; a família como base para o desenvolvimento emocional ao mesmo tempo que é palco de conflitos, o que pode ser a causa ou intensificar sofrimentos psíquicos; e o papel de cuidador, muitas vezes associado a maiores níveis de estresse e sobrecarga pela alta demanda de cuidado e a menores índices de saúde mental, o que impacta diretamente a qualidade de vida e bem-estar do familiar^(4,6,9)^.

Em países da Europa Ocidental, as dinâmicas familiares tendem a excluir a pessoa idosa, o que reforça o fenômeno da transinstitucionalização, caracterizado pela transferência do cuidado de uma instituição para outra - em geral, dos hospitais psiquiátricos para casas de repouso, o que reduz as oportunidades desses idosos se beneficiarem de um modelo comunitário de cuidado em saúde mental, o qual possibilita a aquisição de maior autonomia, aprimoramento de habilidades sociais e diminuição de sintomas psiquiátricos^(7)^.

Já no Brasil, para superação da lógica manicomial e promoção da reabilitação psicossocial, foram criadas estratégias de desinstitucionalização, como o “De Volta para Casa”, programa de ressocialização de pessoas egressas de internações^(6)^, e os Serviços Residenciais Terapêuticos (SRTs), casas ou moradias na comunidade, inseridas na RAPS e direcionadas a egressos de hospitais psiquiátricos e de custódia, pessoas com transtornos mentais que necessitam de auxílio para manter uma moradia, que estejam em situação de rua e/ou que perderam laços sociais e familiares^(2,10-12)^. Contudo, a vulnerabilidade da pessoa idosa pode ser agravada no contexto dos SRTs pois, de forma isolada, esses dispositivos não conseguem responder às múltiplas demandas dessa população, o que evidencia a necessidade de articulação com os serviços da RAPS e de ações intersetoriais para garantir a integralidade do cuidado^(3,8,13,14)^.

Para comportar a heterogeneidade dos moradores, os SRTs são divididos em dois tipos: I, para pessoas com menor grau de dependência, podendo acolher até oito moradores; e II, para aquelas com maior necessidade de cuidados, comportando dez moradores^(10,11)^. Sugere-se que cada Serviço Residencial Terapêutico (SRT) tenha em sua equipe, ao menos, cinco cuidadores e um técnico de enfermagem a cada dez moradores^(11)^ e esteja vinculado a um serviço/equipe de saúde mental de referência, responsável pelo suporte técnico multiprofissional^(11)^. Como atribuição, os profissionais devem auxiliar os moradores na reapropriação do espaço do morar, na construção de habilidades para a vida diária (autocuidado, alimentação, higiene, entre outras), no desenvolvimento de formas de comunicação e no aumento das condições para estabelecimento de vínculos afetivos, de acordo com os respectivos projetos terapêuticos individuais, com foco na inserção na rede social existente e em consonância com a política de reabilitação psicossocial^(2,4,10,11)^.

Uma possibilidade de atuação da equipe multiprofissional, nesse cenário, é o cuidado centrado na família, um dos pilares do modelo comunitário de atenção à saúde mental no âmbito da desinstitucionalização que tem como benefícios à pessoa em sofrimento psíquico melhor funcionamento geral, social e na comunicação, maior adesão ao tratamento, aumento da qualidade de vida, além de ser fonte de segurança e apoio, o que, como consequência, pode reduzir sintomas positivos e negativos do transtorno mental^(15)^.

Estratégias de desinstitucionalização como os SRTs simbolizam a mudança ocorrida no modelo de cuidado brasileiro^(4,12)^. Esses pontos de atenção da RAPS representam uma nova possibilidade de viver em liberdade, ao acolher pessoas vulneráveis, egressas de longas internações e com vínculos familiares fragilizados^(2,5,11)^. No cotidiano do morar, o cuidado em saúde mental permeia as nuances da família, desde os benefícios proporcionados à pessoa idosa em sofrimento psíquico até a sobrecarga vivida pelos familiares enquanto seus cuidadores^(6,9,15)^. Dessa forma, diante do fenômeno da transinstitucionalização, da complexidade de cuidar do idoso e da importância dos SRTs, há uma lacuna de conhecimento no que tange às singularidades das relações familiares no cuidado da equipe multiprofissional nesses dispositivos de desinstitucionalização no modelo comunitário de atenção a saúde mental^(7,13,14)^, principalmente diante de um movimento de retrocesso, em virtude do atual fortalecimento de práticas manicomiais^(16)^.

Por fim, este estudo justifica-se pelo importante papel dos SRTs no contexto da RPB como dispositivos de base territorial para egressos de hospitais psiquiátricos, que tem como propósito principal fornecer moradia e desenvolver o cuidado na perspectiva da reabilitação psicossocial^(2,4,10,11)^, e pela problemática enfrentada pelos profissionais da equipe multiprofissional ao considerar a influência do contexto familiar dos moradores idosos, muitas vezes associado ao desenvolvimento de sofrimentos psíquicos, marcado pela quebra de vínculos, pelo estigma, exclusão e adoecimento dos familiares^(1,2,4,6,9,13-15)^. Portanto, tem-se como objetivo compreender as implicações da família na experiência de cuidado da equipe multiprofissional ao idoso morador de SRTs.

## MÉTODO

### desenho do Estudo

Trata-se de uma pesquisa qualitativa fundamentada na abordagem teórico-metodológica da fenomenologia de Alfred Schutz, a qual almeja apreender o fenômeno a partir da ação social, considerada como um ato intencional moldado pelas motivações individuais dentro do mundo-vida^(17)^. Esse é compreendido como um cenário intersubjetivo habitado pelo homem, em que ele desenvolve suas ações, interações e lhes confere significados, sendo influenciado pelas interações subjetivas face a face e por uma trajetória biográfica, que, juntas, compõem o acervo de conhecimento^(17)^. Schutz propõe compreender os motivos das vivências anteriores (“motivos-porque”) e das expectativas futuras (“motivos-para”) das pessoas no cenário intersubjetivo que habitam e que estabelecem interações por meio da relação face a face, constituindo o seu mundo-vida^(17)^. A partir das motivações, constrói-se o tipo vivido da pessoa no mundo social e são concebidos padrões comuns de significados que auxiliam na compreensão e interpretação da realidade vivida^(17)^. Foram seguidas as recomendações do *Consolidated criteria for reporting qualitative research - COREQ* ^(18)^.

### Local

O estudo foi desenvolvido em um município do interior do estado de São Paulo, que conta com uma Rede de Atenção Psicossocial (RAPS) composta por serviços como leitos de internação psiquiátrica em hospital geral, Centro de Atenção Psicossocial (CAPS) III, CAPS Álcool e Drogas, CAPS Infantojuvenil, SRTs, dentre outros^(19)^. Os SRTs são vinculados aos CAPS III do território onde estão inseridos e se organizam da seguinte forma: quanto à equipe, nas equipes tipo II há profissionais de nível médio (cuidador em saúde ou monitor) disponíveis 24 horas por dia e técnicos de enfermagem, enquanto nas equipes tipo I há um trabalhador de nível médio atuando apenas em um período do dia^(11)^. Três CAPS III do município aceitaram participar da coleta de dados, sendo dois localizados no distrito de saúde Sudoeste e um no Noroeste^(19)^. Eles são responsáveis pelo acompanhamento de cinco SRTs tipo I e dois tipo II, abrigando em torno de 18 moradores no total.

### Participantes

Participaram deste estudo profissionais da equipe multiprofissional que atuam nos CAPS III e desenvolvem cuidados diretos aos idosos dos SRTs, dentre eles enfermeiros, psicólogos, terapeutas ocupacionais, assistentes sociais, técnicos de enfermagem, monitores e auxiliares de moradia. Utilizou-se a amostragem em bola de neve, na qual um participante indica o outro para acesso aos profissionais^(20)^. Os três gestores de cada CAPS III referenciados como sementes, ao indicarem os primeiros participantes que consideravam ter vivências relacionadas à temática do estudo, favorecendo assim a construção de uma rede de confiança e credibilidade ao longo do processo^(20)^. O contato prévio, o convite para participar e o agendamento das entrevistas foram feitos tanto pessoalmente, durante visitas do pesquisador ao serviço, quanto via mensagem eletrônica por aplicativo de celular, com o apoio dos gestores.

Foram considerados os seguintes critérios de inclusão: pertencer à equipe multiprofissional que presta cuidados a moradores idosos dos SRTs, ter pelo menos seis meses de experiência e não estar de férias e nem afastado do trabalho no período da coleta de dados. Não participaram do estudo profissionais que não prestam cuidados diretos aos moradores, como farmacêuticos, técnicos de farmácia, equipe de higiene, serviço de vigilância e auxiliares administrativos.

### Coleta de Dados

A coleta de dados foi realizada de janeiro a dezembro de 2024, por meio de entrevistas fenomenológicas, realizadas pelo primeiro autor, doutorando em ciências da saúde e enfermeiro assistencial à **época**, com vasta experiência acadêmica na metodologia empregada. Tais entrevistas permitem que o participante relate ao entrevistador o sentido atribuído ao fenômeno vivido e à ação realizada em seu contexto de interações sociais^(21)^. As questões disparadoras foram: “Você já cuidou de algum morador idoso no SRT?”, “Conte-me como foi e quais as implicações da família nesse cuidado” e “Qual sua intenção ao realizar esse cuidado?”. A partir delas, foram elaboradas outras questões que permitiram ao participante aprofundar a temática e compartilhar suas vivências. Não foram realizados pré-testes das questões disparadoras, tendo sido definidas com apoio da equipe de pesquisa antes do início da coleta de dados. Ao finalizar a entrevista, foram coletadas informações sobre o tempo de atuação dos participantes nos SRTs e suas experiências de trabalho.

Apenas pesquisa e entrevistado estavam presentes durante as entrevistas, que ocorreram em locais diversos, como nas dependências do CAPS III, nos SRTs e em espaços externos, sendo conduzidas pelo pesquisador principal e gravadas em áudio, com duração média de 25 minutos. Houve uma desistência, justificada por motivos pessoais, momento em que o pesquisador voltou para a semente^(20)^, obtendo nova indicação de participante. Não foram realizadas entrevistas repetidas. A coleta de dados foi encerrada quando o pesquisador principal compreendeu que o fenômeno havia sido desvelado, a inquietação sanada e as ideias nas falas começaram a se repetir, havendo assim suficiência de estruturas de significado subjetivo para descrever o típico da ação. Nesse momento, nenhum novo elemento significativo emergiu nos dados, como nuances, dimensões ou variabilidade^(17,22,23)^.

### Análise e Tratamento dos Dados

Com a finalidade de assegurar o rigor do processo de análise dos dados e compreendendo a fenomenologia como um movimento contínuo, em todas as etapas retornou-se aos principais conceitos de Schutz (ação social, mundo-vida, trajetória biográfica, acervo de conhecimento, relação face a face, motivos-porque e motivos-para)^(17,23)^. Esse retorno constante se justifica porque o processo é mediado pela biografia do pesquisador, exigindo o exercício mental do distanciamento para a adoção de uma atitude científica. Ao interpretar e sistematizar as falas dos sujeitos, elabora-se o tipo vivido, ou seja, a ação de objetivar o subjetivo dos entrevistados^(23)^. Tal elaboração torna-se possível por meio da *epoché* - suspensão dos pressupostos do pesquisador e direcionamento da atenção para suas inquietações e os objetivos do estudo - e da redução eidética, que consiste na análise consciente elaborada a partir das unidades de significação de cada indivíduo^(23)^. Assim, na fenomenologia o conhecimento é construído a partir do vivido no senso comum, sendo que cabe ao pesquisador organizar os dados subjetivos, descrever a vivência e trazê-la para a ordem das significações^(23)^.

A partir do referencial teórico da fenomenologia social^(17)^, os dados provenientes das entrevistas foram analisados pelo pesquisador principal e validados junto ao grupo de pesquisa ao qual ele se encontra vinculado, sem uso de *software*, seguindo as etapas recomendadas em estudos fundamentados na análise fenomenológica e nos conceitos de Schutz^(17,23)^: transcrição na íntegra de cada entrevista, organizando o texto por sujeito a partir da questão norteadora, com o objetivo de aprender o significado individual; leitura e releitura contínua das transcrições para facilitar a apreensão global das vivências dos participantes; recorte das falas que evidenciam as estruturas dos significados subjetivos da ação, reunindo os trechos que revelam os motivos semelhantes; formulação das categorias da ação humana em relação ao significado do fenômeno por meio do agrupamento dos trechos das falas, distinguindo-as em “motivos-porque” - explicam a realização de determinada ação, e “motivos-para” – correspondem as intenções ou finalidades que orientam a ação, conforme ilustração na Figura 1.

**Figura 1:** Diagrama de síntese do processo de análise dos dados.

### Aspectos Éticos

Este estudo foi aprovado pelo Comitê de Ética em Pesquisa da Universidade Estadual de Campinas (UNICAMP), sob o Parecer n.º 4.501.808. Os participantes foram informados de que se tratava de uma investigação científica cuja temática o pesquisador principal vivenciava em seu cotidiano como enfermeiro, buscando assim novas reflexões sobre este tópico para qualificar a prática profissional. Conforme a Resolução nº 466/12, os participantes assinaram o Termo de Consentimento Livre e Esclarecido, o Termo de Autorização para Gravação da Voz e foram informados de que se tratava de uma pesquisa acadêmica. A fim de preservar o anonimato, os relatos foram identificados com a letra “E”, de “entrevistado”, seguida do número correspondente à sequência em que as entrevistas foram realizadas.

## RESULTADOS

Quinze participantes compuseram o estudo, sendo quatro técnicos de enfermagem, quatro monitores, dois enfermeiros, dois terapeutas ocupacionais, um psicólogo, um assistente social e um auxiliar de moradia. O tempo médio de atuação nos SRTs foi de cinco anos. Quanto à experiência de trabalho, para além da atuação em saúde mental, os participantes também foram ou ainda eram: cuidadores de idosos em *home care* e casas de repouso; vinculados a outras instituições de saúde; ou profissionais de setores não associados diretamente à saúde, como empregada doméstica, atendimento ao público e educação.

A partir das entrevistas foi possível organizar os resultados em duas categorias que constituem o tipo vivido dos participantes no mundo social por meio das motivações, descritas a seguir: “A família na experiência de cuidado da equipe multiprofissional ao idoso morador de Serviços Residenciais Terapêuticos”, que caracteriza os “motivos-porque” do significado intersubjetivo das vivências dos participantes no mundo-vida e as implicações familiares no cuidado ao idoso no SRT na perspectiva dos profissionais; e “Intencionalidades da equipe multiprofissional diante da família na experiência de cuidado”, para descrever os “motivos-para” do cuidado realizado pela equipe enquanto ação social, as intencionalidades e o que esperam de suas ações a partir dos encontros face a face.

### A Família na Experiência de Cuidado da Equipe Multiprofissional ao Idoso Morador de Serviços Residenciais Terapêuticos

A equipe multiprofissional identifica que os moradores idosos de SRTs muitas vezes não possuem suporte familiar para a prestação de cuidados e que poucos são os casos em que haveria possibilidade de reconectar esse morador com a família, que por vezes opta por encaminhar o idoso a clínicas de repouso. Nomeiam tais aspectos como abandono e rejeição, reforçados pela dificuldade de estabelecer contato com os familiares, os quais se afastam do cuidado por não conseguirem dar conta de um indivíduo dependente e devido a histórias de conflito familiar.

*Boa parte deles não tem um suporte familiar. [...] Quando ele está na moradia, vai ter todo o suporte que em casa não tem. [...] Dos moradores que a gente tem hoje, que têm contato com a família [...] três ou quatro, assim, eu estou falando um número muito alto, conseguiria voltar para a família. – (E6)*

*Grande parte deles não tem família, e se tem a família, ao longo do tempo, já não presta os cuidados, não tá ali próximo. Então, eu acho legal esse projeto, e uma residência terapêutica, sabe? Grande parte, por alguma questão, ou abandono [...] a família acaba botando em uma clínica, porque fala que não vai conseguir cuidar, porque antes ele andava, ele era independente, agora a gente não consegue cuidar, vai pôr numa clínica. [...] No caso da residência terapêutica, já são os pacientes que não têm família. – (E11)*

*Hoje esse distanciamento familiar, eu já tentei várias aproximações da filha e ela nem me retorna, não atende minhas ligações, ela não quer saber da mãe. [...] Porque muitas vezes também tem essa história do idoso com os familiares, esse idoso a gente não sabe como foi. Tem questão de abandono, rejeição. E quando ele chega nessa idade, essa pessoa que passou por tudo isso, precisa passar por todos esses sentimentos e dar conta. Muitos não conseguem, então não quer contato. [...] A maioria dos idosos tem que lidar com essa questão do abandono, e eu percebo que ela tenta ficar muito mais fora, porque a realidade dói muito. – (E15)*

No contexto de desinstitucionalização proposto pela RPB, muitas vezes o apoio e os vínculos sociais e familiares dos egressos de leitos psiquiátricos estão desgastados, e o SRT surge como alternativa de moradia para que essas pessoas não morem nas ruas, sujeitas a violências e vulnerabilidades. O serviço é tido como uma potencialidade que preza pelo morar, onde os residentes são assistidos e amparados. Entretanto, para os participantes o SRT deve ser considerado apenas quando esgotados os investimentos no núcleo familiar.

*Com todo esse projeto da reforma, do fechamento de leitos e da desinstitucionalização, a gente foi pensando coletivamente e criou-se o projeto da residência terapêutica para pacientes egressos de longos períodos de internação, que não tinha mais vínculo social, não tinha mais vínculo familiar e que não iam mais morar dentro dos hospitais e iriam ter que morar em algum lugar. [...] Núcleos familiares com muito desgaste, onde as relações da pessoa que é a paciente e da rede familiar é muito desgastada [...] indicar para ele ser inserido no programa da residência terapêutica, ou esse paciente ia acabar morador de rua ou sujeito* às *violências e vulnerabilidades sociais e ia piorar a situação de vida dele. [...] A gente tem que investir o que é possível nesse núcleo* (familiar) *para que esse paciente consiga voltar a conviver bem e morar nesse núcleo. Se não há possibilidade, depois de esgotar todos os investimentos, a gente pensa na residência terapêutica. – (E4)*

*Eles nunca tiveram uma casa, eles não tiveram esse apoio, esse cuidado, eles se estranham. [...] Eu estou falando de alguém com mais de 60 anos, que não tem um apoio familiar. Não sabe o que é ir caminhar numa praça, num parque, porque ele nunca teve essa possibilidade. Eu acho que isso é um potencial enorme. Eu acho que a gente consegue ofertar como CAPS, como moradia, eu como referência da casa também, prezar por esse morar. – (E10)*

*Porque são moradores que não têm família, estão envelhecendo, e eles são muito bem cuidados, são assistidos, então, eu acho que eles não estão desamparados. – (E8)*

Em alguns casos, os profissionais relatam boas experiências com moradores idosos do SRT que ainda mantêm vínculos com o núcleo familiar, que os familiares frequentam a moradia para visita, o que contribui para o restabelecimento da autonomia da pessoa. Quando não é esse o caso, os moradores, como parte da reabilitação psicossocial, buscam outras formas de família que não sejam consanguíneas, como quando a comunidade entende e se insere no contexto de vida desses idosos por meio de doações e quando eles próprios se cuidam e se protegem.

*A gente tem boas experiências sobre esse processo de pacientes que hoje ainda mantêm núcleo familiar e a família vai na casa, visita, vai no dia do aniversário, participa dos parabéns, mas que ele vive na residência terapêutica e a família vive lá no espaço dela. Isso foi o que fez o paciente, inclusive, conseguir restabelecer um processo de vida com mais autonomia. – (E4)*

*Eu fico pensando na reabilitação, ela vai desde a questão financeira até o vínculo familiar, que muitas vezes é rompido, mas a gente fica com essa questão familiar mais sanguínea, e eles criam outras famílias, outras redes, que também agrega muito, e eu acredito que isso está dentro da reabilitação. [...] As festas é 100% de doação. Eu acho fantástico, porque é a padaria, o restaurante que doa, o mercado, o bar, as famílias. A vizinha que trabalha na granja fala assim: “tô sabendo que vai ter festa. Você passa lá e pega duas cartelas de ovos”. Então, isso é se apropriar. Isso é a gente fazer com que a comunidade olhe e entenda. – (E15)*

*Quando elas saem do portão, uma cuida da outra. [...] Elas se dão a mão, elas se ajudam [...] Sendo que dentro de casa elas brigam por qualquer coisa, e lá fora, não, lá fora, elas se protegem. Acho que, assim, na cabecinha delas, é a minha família. Então, eu vou cuidar, e elas se cuidam muito bonitinho. – (E2)*

Por fim, a equipe multiprofissional, para além de muitas vezes se considerarem a família dos moradores idosos do SRT, por aproximarem o cuidado ofertado às vivências pessoais, também acreditam que eles a veem como familiares, pois colocam a equipe neste lugar, querem ficar próximos o tempo todo e demonstram gratidão, respeito e carinho pelos profissionais. Como resultado, ao mesmo tempo que sofrem pelo falecimento de moradores idosos, pois passam mais tempo no serviço do que na própria casa, também consideram que o residente gostaria de ter alguém realmente da família em momentos de fragilidade e doença, porque acreditam que não é possível ocupar esse lugar enquanto profissionais.

*Minha mãe era acamada e já aprendi um pouco, então eu vim pra cá já aprendendo já cuidar de uma pessoa idosa. – (E1)*

*Muitos têm família, outros têm ninguém… A família deles, eles veem a gente como sendo a família deles, né? Então, no momento que você chega lá, quando você fica muito tempo sem ir, o dia que você aparece, eles vêm contando as coisas. [...] E você vê que eles querem estar próximos, o tempo todo. – (E3)*

*Acho que de todas as escalas, os sítios que a gente chama, a moradia é o mais importante. Porque na ambiência os pacientes vêm, permanecem na ambiência e vão embora para suas casas… Na residência, não. O que eles têm de base de família, vamos dizer assim, é o pessoal, os monitores, auxiliar de moradias, é a gente que vai lá medicar eles. Acho que a família deles é a gente. Eles não têm grandes tensões. Embora alguns tenham um contato ainda com a família, uma pessoa longe assim, mas eu acho que é isso. – (E6)*

*Isso me faz caminhar na saúde mental sempre, essa gratidão que eles nunca receberam. Essa família que eles não têm, a gente tenta prestar, mas a gente precisa ter esse olhar pro idoso que é muito além. – (E10)*

*Elas olham com carinho. Têm muito respeito pela gente. Tem umas que falam assim..“Ai eu lembro da minha mãe. Que cuidava assim, né?” [...] E eu acho muito triste também quando falece. [...] É como se a gente tivesse perdido alguém da família da gente. Porque a gente está mais aqui do que na casa da gente. [...] Eles têm a gente como se fosse família. [...] Agora eles, acho que não pensam assim. Queria naquela hora que está ruim... A família, alguém do lado, não tem ninguém aí. Aí que pega na gente. [...] – (E7)*

*Também não dá pra gente ir fantasiando e tentar ocupar outros lugares da história deles, que acho que é sobre isso também, acho que a saúde mental tem disso, dessa sensibilização, dessa reaproximação, da vida e com a família e tudo mais, mas também faz parte da história deles. – (E5)*

### Intencionalidades da Equipe Multiprofissional Diante da Família na Experiência de Cuidado

A partir da ideia de considerar os moradores idosos como membros da família, a equipe intenciona realizar o cuidado da melhor maneira, com carinho e da forma como gostaria que fosse realizado com eles. Nos casos em que eles possuem rede familiar, identifica-se a importância do profissional de referência articular o cuidado junto à família, para além dos demais serviços da RAPS em que o morador faz acompanhamento.

**É** *bom, eu gosto e procuro cuidar com o maior carinho, como se fosse um dos meus, da minha família. Então eu procuro fazer o melhor, como eu quero ser tratada, eu procuro tratar o próximo. – (E12)*****

*As discussões das moradias precisam estar dentro do CAPS. A referência precisa estar junto com o enfermeiro da miniequipe do CAPS, junto com o médico. A referência precisa articular esses cuidados com a rede. Então, se é com a UNICAMP, com o Mário Gatti, com o centro de saúde, com a família, porque tem alguns que têm família, outros não. E a gente poder acompanhar nesses* últimos *momentos da vida. – (E14)*

A equipe gostaria que os moradores idosos tivessem família, para que pudessem ficar juntos e terem alguém para visitar, apesar das dificuldades de conviver com ela. A partir disso, identificam que os profissionais do SRT são a família desses moradores.

*Eu gostaria que eles tivessem família, todos. Eu acho, assim, que por mais difícil que seja você viver com a família, não existe coisa, sentimento igual. Então, assim, eu acho que a primeira coisa era eles terem alguém por eles, sabe? A família, eles poderem estar juntos, ir na casa, todos irem. Sair, poder ir… Eu fui na minha mãe, eu fui na minha tia, eu fui em alguém… Fui na casa de alguém, de um familiar. – (E3)*

*A família não acompanha. A gente acaba sendo a família pra eles. É o que me faz continuar. – (E13)*

Um dos membros da equipe relatou uma experiência singular durante sua formação, quando conheceu serviços de moradia em outro país, modelo no qual as casas são distribuídas pelo município e a equipe vai até a residência a partir da demanda dos moradores, vislumbrando esse funcionamento para os SRTs no Brasil.

*Tive a experiência na residência de ir para Portugal e conhecer o Housing First, por exemplo, que é outra lógica. A gente sabe que a gente está falando de outro país, outra cultura, outra política pública. Então, não dá para a gente querer comparar, copiar e colar o projeto para cá. [...] Talvez eu vislumbre ter essa experiência aqui. Lá são casas individuais, ou um indivíduo mesmo ou famílias têm as suas próprias casas distribuídas pelo território do município e eles faziam através de visitas ou através de demanda. [...] Então, para fazer a limpeza da casa, compra de mercado, organização financeira,* às *vezes, algum acompanhamento com um serviço de saúde. Então, tinham profissionais que ficavam referências das casas espalhadas. Eu, enquanto referência, teria cinco casas responsáveis. Mas teria a semana para esse cuidado. – (E9)*

## DISCUSSÃO

A aproximação com a experiência vivida pelos profissionais que compõem a equipe multiprofissional permitiu compreender alguns aspectos relacionados às implicações da família na experiência de cuidado ao idoso morador de SRTs. As ações sociais dos participantes desvelaram as motivações existentes no seu mundo-vida, em que as relações com os moradores idosos no cotidiano do cuidado no SRT constroem o espaço intersubjetivo que permite agregar conhecimento e pavimentar a trajetória biográfica^(17)^.

Um dos principais achados deste estudo versa sobre inexistência ou fragilidade de suporte familiar para os moradores idosos de SRTs. A composição familiar é particularmente importante, tendo em vista que a família é tida como a unidade social mais básica na vida humana e parte do mundo-vida das pessoas, intimamente relacionada ao fornecimento de apoio físico, psicológico e espiritual, além de ofertar cuidados diários. Na população idosa, são comuns os sentimentos de solidão e abandono, questões complexas, com profundas implicações no bem-estar e na saúde mental e que requerem abordagens integradas e articulações intersetoriais que considerem aspectos individuais, relacionais e contextuais^(24)^.

Ressalta-se que pessoas idosas são vulneráveis nos âmbitos individual, social e programático^(8)^. Aquelas egressas de longas internações psiquiátricas estão mais propensas à perda de vínculos familiares ou à fragilidade desses laços^(10)^. Essa realidade, associada às consequências do modelo manicomial, constitui um dos critérios para ingresso nos SRTs^(11)^. Assim, a fragilidade ou inexistência de suporte familiar entre os moradores idosos desses dispositivos revela uma ruptura significativa em seu mundo-vida. Nesse sentido, morar em um SRT não significa somente ter cuidado e moradia, mas representa uma resposta das políticas públicas diante da vulnerabilidade programática dos idosos^(8)^, além de possibilitar a reconstrução de redes intersubjetivas e a ressignificação de trajetórias^(17)^.

O cuidado familiar está inserido no mundo-vida e as pessoas que o recebem possuem melhor prognóstico de saúde e qualidade de vida^(6)^. Contudo, a carga e a responsabilidade atribuídas aos familiares também estão associadas a altos níveis de sofrimento psíquico^(6)^. O cuidado de um idoso com psicose, por exemplo, em que comorbidades orgânicas são comuns, desafia o estoque de conhecimento disponível, exige mais e é mais estressante, uma vez que rompe com tipificações associadas ao envelhecimento considerado típico^(6)^. Aponta-se que o familiar cuidador do idoso em sofrimento psíquico vivencia o desgaste emocional, a dificuldade de conviver com os sintomas comportamentais e identifica a necessidade de dividir a responsabilidade de cuidar com os serviços de saúde na tentativa de aliviar a sobrecarga^(25)^. Portanto, o adoecimento psíquico dos cuidadores devido a sobrecarga de cuidado pode, inclusive, afetar sua capacidade de cuidar, sua qualidade de vida e sua autoestima, e fazer com que tenham comportamentos hostis, em um ciclo vicioso que pode desestabilizar o tecido relacional que sustenta o mundo social, gerar conflitos familiares e impactar negativamente a saúde da pessoa cuidada^(6)^.

Apesar de ser dever da família o amparo à pessoa idosa, nem sempre os familiares possuem o acervo de conhecimento para o cuidado necessário durante o processo de envelhecimento, com suas modificações físicas e psicossociais no mundo-vida^(26)^. Essa situação se intensifica na sociedade atual, em que as famílias são cada vez menos numerosas, o ritmo de trabalho é mais intenso e o cuidado, quase sempre, recai para um integrante da família^(26)^. Nesse contexto, ao tensionar a tipificação tradicional do papel familiar, é comum a delegação do cuidado para instituições de longa permanência, por exemplo, sendo essas novas redes intersubjetivas muitas vezes consideradas pelos familiares como um ambiente mais adequado para suprir as necessidades de saúde do idoso^(26)^. Entretanto, pessoas idosas com sofrimento psíquico grave apresentam elevada vulnerabilidade e necessidades específicas de cuidados prolongados e frequentemente enfrentam barreiras significativas na admissão em instituições de longa permanência, que, em geral, têm dificuldades para oferecer suporte especializado em saúde mental, o que compromete a qualidade do cuidado^(27)^, reforça o fenômeno da transinstitucionalização^(7)^ e evidencia um importante desafio ainda não superado^(27)^.

Para idosos em sofrimento psíquico com vínculos familiares e laços sociais fragilizados, o SRT se mostra como alternativa de espaço que preza pelo morar, ao proporcionar um ambiente intersubjetivo para o desenvolvimento de relações sociais e afetivas para aqueles que até então se encontravam confinados em hospitais psiquiátricos, tendo como principal objetivo o resgate da autonomia e o direito de viver em comunidade por meio da reabilitação psicossocial. Assim, as pessoas, que antes eram tidas como pacientes, no SRT são moradores^(2,28)^ e, a partir do seu acervo de conhecimentos, podem reconstruir novos percursos em seu mundo-vida^(17)^. O cuidado em regime manicomial tinha como marcas biográficas as limitações de espaço e de individualidade, transformadas ao morador do SRT em possibilidades de circular na comunidade, conviver e interagir com outras pessoas por meio de relações face a face, trocar experiências e, principalmente, aprender com o outro e com o mundo social que o cerca^(17,28)^. Nesse processo, a ação da equipe multiprofissional é fundamental para estimular o viver no território dos moradores dos SRTs, o exercício da cidadania e a configuração desse espaço como um lar e não um local de tratamento^(29)^.

Além disso, o sentimento de pertencer e habitar um espaço possui grande significado às pessoas em sofrimento psíquico, uma vez que o morar se constitui no mundo-vida como construção intersubjetiva carregada de sentidos^(17)^ e “casa”, comumente, é local de acolhimento, conforto, intimidade, trocas e vivências de relações afetivas e interpessoais a partir do acervo de conhecimento, sendo legitimadas por meio do cotidiano doméstico entre os moradores idosos do SRT^(28)^. O espaço de moradia deve se adaptar às intencionalidades de seus moradores e fornecer um suporte flexível por parte dos profissionais, para além de padrões externos de organização, pois o “estar em casa” representa a oportunidade de expressar suas subjetividades e o exercício da vida em liberdade^(13)^. A partir disso, constrói-se um novo entendimento do “morar”: agora uma construção subjetiva a partir da biografia de cada morador, que possui um lugar para chamar de “seu” no mundo-vida^(2,13,28)^.

Em alguns casos, ainda se identifica a existência de vínculos entre o morador idoso e seus familiares, que fazem visitas ao SRT. O modelo manicomial produz exclusão e distanciamento do mundo social, com impacto nas relações familiares desde as primeiras internações psiquiátricas, pois estas pessoas costumam não receber visitas no tempo em que permanecem nas instituições, o que prejudica as relações sociais e de afeto^(13)^. A interação frequente entre família e idoso por meio de visitas ao SRT, nos casos em que não vivem juntos, não supre as necessidades de comunicação interpessoal do morador. Contudo, essa aproximação ainda assim favorece a saúde física e mental, atualiza os estoques de conhecimento e reforça o sentimento de pertencimento e continuidade biográfica no mundo-vida, além de possibilitar uma percepção mais positiva do processo de envelhecimento, o que impacta a qualidade de vida^(24)^. Conforme apontado pelos participantes, tais visitas são caracterizadas como encontros face a face e representam impactos positivos em oposição à exclusão típica das práticas manicomiais, o que pode auxiliar na motivação das ações futuras do morador e da equipe multiprofissional^(17)^.

A fim de preencher a lacuna das relações familiares frágeis ou inexistentes, os moradores idosos buscam outras formas de família, seja dentro ou fora do SRT, em um movimento de ocupação do território, reconstrução do pertencimento no mundo-vida e atualização do estoque de conhecimento^(17)^. Ao morar em uma mesma residência, os idosos desenvolvem a sensação de pertencimento e do habitar em coletivo, ressignificando as tipificações familiares e ampliando as relações afetivas, intersubjetivas e sociais^(17,28)^. No contexto da reabilitação psicossocial, a ação de morar também se torna uma forma de apropriação material e simbólica dos espaços da casa, onde a convivência se consolida como um processo de troca de recursos, cuidados e afetos entre os moradores^(28)^. Entretanto, há uma centralidade dos SRTs na rede de serviços de saúde, o que pode restringir a reinserção social dos moradores e dificultar a criação de novos laços^(13)^. Tal fato aponta para a necessidade de se pensar as ações de cuidado em uma perspectiva intersetorial^(3,8,13)^.

Além disso, a superação do modelo manicomial exige o envolvimento dos moradores idosos na cidade, tendo em vista a porosidade do tecido urbano proporcionada pelos SRTs, por meio da inserção em grupos sociais e/ou espaços públicos e comunitários, o que promove a ampliação do mundo-vida e a ressignificação de tipificações sociais associadas à exclusão^(17,28)^. Essa forma de participação social fortalece a intersubjetividade e pode transformar o cotidiano, o percurso biográfico e as condições de vida do idoso, que muitas vezes são marcadas por adoecimento, violência, injustiça social, desigualdade, preconceitos, exclusão e opressão^(28)^. Na reinserção social, as atitudes da comunidade são fundamentais, principalmente ao buscarem promover a conscientização sobre a saúde mental por meio do contato direto com os moradores dos SRTs, ações que ajudam na redução do estigma e no fortalecimento do modelo de cuidado em liberdade^(30)^. Como consequência do viver em sociedade, os moradores expandem sua comunicação interpessoal, cultivam *hobbies* e melhoram sua qualidade cultural ao valorizarem a subjetividade e a potencialidade dos encontros^(28)^.

Nesse contexto, os próprios profissionais se consideram família dos moradores idosos. A autonomia no SRT, quando comparada às instituições asilares, é superior devido principalmente à construção de interdependência entre os dispositivos, território e pessoas, sendo nesse último caso também considerada a relação face a face entre moradores e profissionais para produzir novos sentidos^(28)^. A construção dessas relações é parte integrante do processo de desinstitucionalização, uma vez que a possibilidade de fazer escolhas permite a reconstrução biográfica e ressignificação dos sentidos e estoques de conhecimento do seu mundo-vida^(17,28)^. Além disso, as ações dos profissionais no cotidiano vão muito além das questões físicas e psicossociais. Trata-se de uma perspectiva de cuidado relacional e terapêutico, expresso por meio da escuta qualificada, da comunicação interpessoal e da construção de vínculos com novos significados no mundo-vida, que também propiciam conforto emocional e espiritual^(17,31)^. Essa interação revela o potencial da abordagem multiprofissional em saúde mental mediante o cuidado centrado na pessoa e na valorização da subjetividade^(31)^, aspectos fundamentais para a reabilitação psicossocial.

No entanto, é preciso haver cuidado por parte dos profissionais que, ao considerarem o morador idoso como um familiar, demonstram ambiguidade de suas funções social e terapêutica, necessárias para sua atuação no SRT^(2,10)^. Essa atitude de cuidar associa-se à caridade, restringe a intersubjetividade e reproduz a ideia de isolamento social perpetuada historicamente nos manicômios, uma vez que se faz necessário ressignificar o cuidado de maneira a considerar que esses dispositivos necessitam de intersetorialidade e de conhecimento técnico, alinhando-os às motivações orientadas para fortalecimento e construção do mundo-vida dos moradores idosos^(2,10,17)^. Embora existam, as ações intersetoriais no âmbito do cuidado na RAPS ocorrem por meio de atendimentos compartilhados e reuniões, muitas vezes de modo restrito e isolado, sem uma sistematização efetiva^(3)^. Esse contexto reflete uma rede fragmentada e centralizada nos serviços especializados, mas que necessita de articulações fortalecidas entre níveis e serviços para garantir o cuidado longitudinal, territorial e baseado no conhecimento técnico^(3)^. Sendo assim, a compreensão fenomenológica contribui, ao desvelar que a ação de cuidar da equipe multiprofissional está permeada por uma atitude caridosa, característica associada à vivência prévia das pessoas e com forte apelo social, o que pode se distanciar de práticas amparadas em conhecimento técnico e científico^(17)^.

Assim, é possível destacar que o modelo atual de cuidado em saúde mental ainda enfrenta obstáculos na efetivação do cuidado em liberdade no território^(3,13,29)^. Propostas que incentivam a internação psiquiátrica e o financiamento de comunidades terapêuticas baseadas em uma visão proibicionista e excludente da pessoa em sofrimento psíquico ainda persistem^(16)^. Tais ações refletem tentativas de retomada do modelo manicomial, o que reforça a importância de reflexões críticas sobre as práticas nos serviços de saúde^(13,16)^. Nesse contexto, destaca-se a ação dos profissionais, cuja atuação deve estar alinhada com os princípios da reabilitação psicossocial e da garantia de direitos, a fim de superar lógicas institucionais ainda vigentes^(13)^.

Os profissionais relatam realizar o cuidado aos moradores idosos do SRT da forma como gostariam que fosse realizado com eles, o que demonstra empatia. A empatia é um construto multidimensional que inclui fatores cognitivos e afetivos, sendo considerada como a compreensão, por parte do profissional, dos sentimentos do morador, aspecto essencial em contextos de saúde mental, na qual pessoas em sofrimento psíquico valorizam a perspectiva dos profissionais e seu entendimento sobre a situação^(32)^. A empatia exercida pelos profissionais do SRT emerge do mundo-vida compartilhado e das relações face a face, permitindo compreender as experiências dos moradores a partir de seus estoques de conhecimento e das tipificações do vivido^(17)^. A capacidade empática também permite adotar posições não defensivas e facilita a obtenção de resultados satisfatórios e produtivos^(32)^. Contudo, no contexto do cuidado em saúde mental, a empatia não pode ser vista apenas como uma característica pessoal, mas como uma competência relacional que se torna elemento central da relação terapêutica^(31)^, possibilitando escuta qualificada, reconhecimento da singularidade e fortalecimento dos vínculos com os moradores dos SRTs. Assim, a empatia pode ser compreendida como uma perspectiva que deve permear a atuação de toda a equipe multiprofissional, como um aspecto essencial do cuidado^(32)^.

Os participantes identificam a importância em articular o cuidado junto aos familiares e demais serviços em que o morador idoso faz acompanhamento, o que representa um processo de ampliação da intersubjetividade e de atualização dos estoques de conhecimento sobre as singularidades dos moradores, e demonstra a necessidade dos profissionais do SRT realizarem orientação, educação e apoio aos familiares e trabalhadores de outros contextos de saúde^(33)^. Essa prática pode ser compreendida como uma estratégia de enfrentamento da vulnerabilidade programática da pessoa idosa e da fragmentação da rede de serviços, ao possibilitar que os profissionais articulem ações para ampliar o acesso, fortaleçam os vínculos e assegurem a continuidade da atenção à saúde^(3,8)^. Como consequência, pode haver uma reaproximação do morador com o contexto familiar e outros espaços da comunidade, a fim de contribuir para a participação social e a reorganização do mundo-vida dos idosos, em consonância com projetos orientados à autonomia e à inclusão^(28)^.

Outros apontamentos dos profissionais foram de que gostariam que os moradores idosos tivessem família e, por isso, se colocam no lugar de familiares. Na vertente da empatia, essa é importante fator para o desenvolvimento da relação entre morador e profissional, ao passo que estreita as relações face a face e a confiança para o cuidado, e é preciso haver cautela para não ultrapassar os limites dos papéis sociais tipificados, fundamentais para a clareza das funções no SRT^(2,10,32)^. Como alternativa, em consonância com a reabilitação psicossocial, o profissional pode, a partir dos estoques de conhecimento dos moradores, ajudar a resgatar, reconhecer e validar os desejos do idoso de reencontrar amigos, familiares, lugares e relações que vivenciou ao longo de sua vida antes da internação psiquiátrica, com o objetivo de reviver momentos de felicidade e proteção^(28)^.

Por fim, os profissionais identificam o desejo de que o cuidado ao público do SRT seja realizado fora de instituições, como por exemplo por meio de visitas em residências espalhadas no território, o que revela as ações orientadas para motivação futura que visa à construção de práticas alinhadas ao mundo-vida do morador^(17)^. Essa ideia alinha-se a iniciativas ao redor do mundo, como o *Housing First*, que, ao contrário do movimento de transinstitucionalização evidenciado internacionalmente^(7)^, prioriza a moradia imediata e independente, valoriza a autonomia e a escolha da pessoa^(34)^, características que visam cada vez mais o cuidado fora de hospitais e de instituições comunitárias, realizado na própria casa da pessoa, quando possível^(35)^. Para isso, é preciso que os profissionais de saúde se tornem agentes ativos nas discussões acerca do isolamento social e das necessidades de saúde mental, principalmente da população idosa^(35)^. Desvela-se que as motivações futuras dos participantes se encontram na intencionalidade de cuidar da saúde mental em liberdade nas distintas fases da vida por meio da valorização da trajetória biográfica, trazendo luz a problemáticas como o isolamento da pessoa idosa em sofrimento psíquico^(17)^.

Este estudo evidencia as práticas da equipe multiprofissional no âmbito das implicações da família no cuidado diário dos idosos moradores dos SRTs, ressaltando a importância dessa dimensão em seu mundo-vida. A abordagem voltada para as singularidades do público idoso nos dispositivos de reabilitação psicossocial aponta para a relevância de se ampliar a discussão sobre as necessidades intersubjetivas dessas pessoas e qualificar os profissionais para o cuidado, entre eles a equipe de enfermagem, tendo em vista o cenário de envelhecimento populacional.

Em relação às limitações do estudo, destaca-se que os participantes são profissionais que atuam em alguns SRTs do município onde a pesquisa foi realizada, estando os resultados vinculados às experiências, às percepções e aos contextos específicos dos sujeitos envolvidos^(17)^. Contudo, este estudo não se propôs a analisar outras características que podem influenciar o mundo-vida e a ação cotidiana nos SRTs. Recomenda-se, que pesquisas futuras explorem outros aspectos estruturais, históricos e sociais, especialmente em relação à participação e ao papel da família no processo de desinstitucionalização e na reabilitação psicossocial de pessoas idosas, ampliando a compreensão sobre os desafios e as potencialidades desse cuidado em diferentes realidades socioculturais.

## CONCLUSÃO

Neste estudo, foi possível construir o tipo vivido a partir das categorias temáticas que evidenciaram as motivações das vivências dos participantes, sendo essas vivências permeadas pelas implicações da família no mundo-vida do morador idoso do SRT, o que influencia no cuidado realizado pela equipe multiprofissional.

A partir dos resultados, nota-se que o percurso biográfico dos idosos moradores do SRT é marcado por vulnerabilidade, ausência, fragilização e rompimento dos vínculos familiares, situando os SRTS como importantes espaços no processo de desinstitucionalização e reabilitação psicossocial. A atuação dos profissionais no cotidiano por meio da escuta qualificada e da empatia possibilita ressignificar experiências de exclusão e de sofrimento dos moradores. Nesse contexto, observa-se que a ruptura das relações no mundo-vida torna os SRTs uma alternativa de moradia e cuidado potente, com possibilidade de acolhimento e reconstrução de vínculos afetivos, intersubjetivos e sociais, seja entre moradores que ainda mantêm contato com suas famílias de origem ou por meio da formação de novos arranjos relacionais dentro da própria casa ou na comunidade. A equipe multiprofissional também reconhece sua função de referência afetiva e articuladora das ações no mundo-vida dos moradores para garantir um cuidado qualificado e humanizado a partir da biografia pregressa e na reconstrução de sentidos na vida cotidiana. Apesar disso, em momentos de maior fragilidade emocional, tanto a equipe quanto os moradores gostariam que houvesse apoio de familiares.

A intencionalidade das ações da equipe multiprofissional nos SRTs é marcada pela busca por um cuidado qualificado aos moradores idosos. Quando há presença de familiares, ainda que permeada por conflitos, a equipe espera que o profissional de referência articule a relação entre eles, somado à expectativa de que os moradores possam contar com suas próprias famílias. Porém, as diferentes formas de organização desses espaços também influenciam as vivências, indicando que o modo como o serviço é estruturado pode potencializar ou limitar a relação com a família.

Além disso, os achados deste estudo apontam a necessidade de o cuidado multiprofissional nos SRTs, principalmente pelos profissionais que ficam mais próximos dos moradores, como a equipe de enfermagem, ser orientado por uma ação ampliada e pela articulação intersetorial, ao reconhecer as singularidades dos moradores na perspectiva de superar práticas fragmentadas nos serviços da rede. Assim, essas ações podem ser compreendidas como estratégias fundamentais no enfrentamento à vulnerabilidade programática das pessoas idosas. Por fim, os anseios expressos pelos profissionais por modelos de cuidado territorializados e integrados revelam a motivação da ação alinhada aos princípios da reabilitação psicossocial, ao exercício da cidadania e ao direito de existir na sociedade.

## DISPONIBILIDADE DE DADOS

Todo o conjunto de dados que dá suporte aos resultados deste estudo está disponível mediante solicitação ao autor correspondente.

# Translation: La familia en la experiencia del cuidado del equipo multiprofesional al anciano residente en Servicios Residenciales Terapéuticos

**DOI:** 10.1590/1980-220X-REEUSP-2025-0254es

**Authors:** Aldair Weber, Giulia Delfini, Joaquim Manuel de Oliveira Lopes, Vanessa Pellegrino Toledo

**Affiliations:** 1 Universidade Estadual de Campinas, Faculdade de Enfermagem, Campinas, SP, Brasil.; 2 Universidade de Lisboa, Escola Superior de Enfermagem, Centro de Investigação, Inovação e Desenvolvimento em Enfermagem de Lisboa, Lisboa, Portugal.; 3 Universidade de Lisboa, Escola Superior de Enfermagem, Lisboa, Portugal.

**Keywords:** Anciano, Grupo de Atención al Paciente, Familia, Rehabilitación Psiquiátrica, Servicios de Salud Mental

## Abstract

**Objetivo:**

Comprender las implicaciones de la familia en la experiencia de cuidado del equipo multidisciplinario a los adultos mayores residentes en Servicios Residenciales Terapéuticos.

**Método:**

Investigación cualitativa basada en la fenomenología social. Se realizaron entrevistas fenomenológicas a 15 profesionales de equipos multidisciplinarios de Servicios Residenciales Terapéuticos de un municipio del interior de São Paulo.

**Resultados:**

Las experiencias en el cuidado de personas mayores en el Servicio Residencial Terapéutico y sus implicaciones familiares caracterizaron los “motivos-por qué” en la categoría “La familia en la experiencia de cuidado del equipo multidisciplinario a personas mayores residentes en Servicios Residenciales Terapéuticos”. Estos dispositivos de desinstitucionalización valoran la convivencia, donde los residentes pueden fortalecer los lazos familiares y establecer nuevas relaciones. La motivación futura se describe en la categoría “Intencionalidades del equipo multidisciplinario frente a la familia en la experiencia del cuidado”.

**Conclusión:**

La desinstitucionalización permite al residente anciano reconstruir vínculos afectivos, asumiendo el equipo el papel de referencia afectiva en el recorrido biográfico de su mundo-vida. La expectativa de articular el cuidado con la presencia de los familiares tiene como objetivo mejorar las experiencias de los residentes ancianos.

## INTRODUCCIÓN

La desinstitucionalización psiquiátrica, pionera en Italia, se considera un hito importante en la modernización de la atención de la salud mental^(1)^. A partir de ella, en Brasil, la Reforma Psiquiátrica Brasileña (RPB) impulsó la reforma sanitaria que dio origen al Sistema Único de Salud (SUS), transformando la política de salud mental en el país^(2,3)^. La Ley n.º 10.216/2001, referencia de la lucha contra los manicomios y de la RPB, garantiza los derechos de las personas con trastornos psíquicos y reorienta el modelo de atención, que pasó a ser de base territorial y comunitaria, estructurado por la Red de Atención Psicosocial (RAPS), cuyo objetivo es crear, ampliar y articular puntos de atención a la salud en el ámbito del SUS^(3-5)^.

El movimiento de desinstitucionalización promovió importantes transformaciones en la atención a la salud en varios países, permitiendo que las personas con trastornos mentales vivieran el proceso de envejecimiento^(6,7)^. En este sentido, la atención a la vejez resulta compleja, ya que las condiciones socioeconómicas desfavorables predisponen a las personas mayores a la vulnerabilidad, con resultados adversos para la salud y un impacto en la calidad de vida^(8)^. La presencia de un mayor déficit funcional, menores ingresos familiares y una mayor dependencia de los servicios públicos caracterizan la vulnerabilidad programática de las personas mayores, una dimensión que debe considerarse como un aspecto importante en la planificación de las acciones de los servicios de salud, al reforzar la necesidad de una atención en red mediante la articulación entre diferentes puntos de atención^(8)^.

Debido a la mayor necesidad de las personas mayores de apoyo emocional, asistencia financiera y realización de actividades de la vida diaria, los familiares a menudo se convierten en cuidadores^(6)^. Al considerar el cuidado de las personas mayores con trastornos mentales graves, es posible enumerar algunos problemas que afectan a la familia: la ruptura de los lazos familiares; la familia como base para el desarrollo emocional y, al mismo tiempo, escenario de conflictos, lo que puede ser la causa o intensificar el sufrimiento psíquico; y el papel de cuidador, a menudo asociado a mayores niveles de estrés y sobrecarga por la alta demanda de cuidados y a menores índices de salud mental, lo que repercute directamente en la calidad de vida y el bienestar del familiar^(4,6,9)^.

En los países de Europa Occidental, las dinámicas familiares tienden a excluir a las personas mayores, lo que refuerza el fenómeno de la transinstitucionalización, caracterizado por la transferencia del cuidado de una institución a otra, en general, de los hospitales psiquiátricos a las residencias de ancianos, lo que reduce las oportunidades de estas personas mayores de beneficiarse de un modelo comunitario de atención de salud mental, que permite adquirir una mayor autonomía, mejorar las habilidades sociales y disminuir los síntomas psiquiátricos^(7)^.

En Brasil, para superar la lógica manicomial y promover la rehabilitación psicosocial, se crearon estrategias de desinstitucionalización, como “De Volta para Casa” (De vuelta a casa), un programa de resocialización de personas que han salido de internamientos^(6)^, y los Servicios Residenciales Terapéuticos (SRT), casas o viviendas en la comunidad, insertadas en la RAPS y dirigidas a personas que han salido de hospitales psiquiátricos y de custodia, personas con trastornos mentales que necesitan ayuda para mantener una vivienda, que están viviendo en la calle y/o que han perdido los lazos sociales y familiares^(2,10-12)^. Sin embargo, la vulnerabilidad de las personas mayores puede agravarse en el contexto de los SRT, ya que, de forma aislada, estos dispositivos no pueden responder a las múltiples demandas de esta población, lo que pone de manifiesto la necesidad de articulación con los servicios de la RAPS y de acciones intersectoriales para garantizar la integralidad de la atención^(3,8,13,14)^.

Para dar cabida a la heterogeneidad de los residentes, los SRT se dividen en dos tipos: I, para personas con un menor grado de dependencia, con capacidad para acoger hasta ocho residentes; y II, para aquellas con mayor necesidad de cuidados, con capacidad para diez residentes^(10,11)^. Se sugiere que cada Servicio Residencial Terapéutico (SRT) cuente en su equipo con al menos cinco cuidadores y un técnico de enfermería por cada diez residentes^(11)^y que esté vinculado a un servicio/equipo de salud mental de referencia, responsable del apoyo técnico multiprofesional^(11)^. Como atribución, los profesionales deben ayudar a los residentes a reapropiarse del espacio de la vivienda, a desarrollar habilidades para la vida diaria (autocuidado, alimentación, higiene, entre otras), a desarrollar formas de comunicación y a mejorar las condiciones para establecer vínculos afectivos, de acuerdo con los respectivos proyectos terapéuticos individuales, con enfoque en la inserción en la red social existente y en consonancia con la política de rehabilitación psicosocial^(2,4,10,11)^.

Una posibilidad de actuación del equipo multiprofesional, en este escenario, es la atención centrada en la familia, uno de los pilares del modelo comunitario de atención a la salud mental en el ámbito de la desinstitucionalización, que tiene como beneficios para la persona que sufre psíquicamente un mejor funcionamiento general, social y en la comunicación, una mayor adherencia al tratamiento, un aumento de la calidad de vida, además de ser una fuente de seguridad y apoyo, lo que, como consecuencia, puede reducir los síntomas positivos y negativos del trastorno mental^(15)^.

Las estrategias de desinstitucionalización, como los SRT, simbolizan el cambio que se ha producido en el modelo de atención brasileño^(4,12)^. Estos puntos de atención de la RAPS representan una nueva posibilidad de vivir en libertad, al acoger a personas vulnerables, que han salido de largas hospitalizaciones y con vínculos familiares frágiles^(2,5,11)^. En la vida cotidiana, la atención de salud mental impregna los matices de la familia, desde los beneficios que se proporcionan a las personas mayores que sufren psíquicamente hasta la sobrecarga que experimentan los familiares como cuidadores^(6,9,15)^. Así, ante el fenómeno de la transinstitucionalización, la complejidad del cuidado de las personas mayores y la importancia de los SRT, existe una laguna de conocimiento en lo que respecta a las singularidades de las relaciones familiares en la atención del equipo multiprofesional en estos dispositivos de desinstitucionalización en el modelo comunitario de atención a la salud mental^(7,13,14)^, principalmente ante un movimiento de retroceso, debido al actual fortalecimiento de las prácticas manicomiales^(16)^.

Por último, este estudio se justifica por el importante papel de los SRT en el contexto de la RPB como dispositivos de base territorial para los egresados de hospitales psiquiátricos, cuyo objetivo principal es proporcionar vivienda y desarrollar la atención desde la perspectiva de la rehabilitación psicosocial^(2,4,10,11)^, y por la problemática enfrentada por los profesionales del equipo multiprofesional al considerar la influencia del contexto familiar de los residentes de edad avanzada, a menudo asociada al desarrollo de sufrimientos psíquicos, marcados por la ruptura de vínculos, el estigma, la exclusión y la enfermedad de los familiares^(1,2,4,6,9,13-15)^. Por lo tanto, el objetivo es comprender las implicaciones de la familia en la experiencia de cuidado del equipo multiprofesional a los ancianos residentes en SRTs.

## MÉTODO

### Diseño del Estudio

Se trata de una investigación cualitativa basada en el enfoque teórico-metodológico de la fenomenología de Alfred Schutz, que pretende comprender el fenómeno a partir de la acción social, considerada como un acto intencional moldeado por las motivaciones individuales dentro del mundo-vida^(17)^. Este se entiende como un escenario intersubjetivo habitado por el hombre, en el que desarrolla sus acciones e interacciones y les confiere significados, influenciado por las interacciones subjetivas cara a cara y por una trayectoria biográfica que, en conjunto, conforman el acervo de conocimientos^(17)^. Schutz propone comprender los motivos de las experiencias anteriores (“motivos-por qué”) y las expectativas futuras (“motivos-para”) de las personas en el escenario intersubjetivo que habitan y que establecen interacciones a través de la relación cara a cara, constituyendo su mundo-vida^(17)^. A partir de las motivaciones, se construye el tipo vivido de la persona en el mundo social y se conciben patrones comunes de significados que ayudan a comprender e interpretar la realidad vivida^(17)^. Se siguieron las recomendaciones de los Criterios consolidados para la presentación de informes de investigación cualitativa – COREQ^(18)^.

### Lugar

El estudio se llevó a cabo en un municipio del interior del estado de São Paulo, que cuenta con una Red de Atención Psicosocial (RAPS) compuesta por servicios como camas de hospitalización psiquiátrica en un hospital general, Centro de Atención Psicosocial (CAPS) III, CAPS Alcohol y Drogas, CAPS Infantojuvenil, SRTs, entre otros^(19)^. Los SRT están vinculados a los CAPS III del territorio en el que se encuentran y se organizan de la siguiente manera: en cuanto al personal, en los equipos de tipo II hay profesionales de nivel medio (cuidadores de salud o monitores) disponibles las 24 horas del día y técnicos de enfermería, mientras que en los equipos de tipo I hay un trabajador de nivel medio que trabaja solo durante un periodo del día^(11)^. Tres CAPS III del municipio aceptaron participar en la recopilación de datos, dos de ellos ubicados en el distrito de salud Sudoeste y uno en el Noroeste^(19)^. Son responsables del seguimiento de cinco SRT tipo I y dos tipo II, que albergan a unos 18 residentes en total.

### Participantes

Participaron en este estudio profesionales del equipo multiprofesional que trabajan en los CAPS III y prestan atención directa a los ancianos de los SRT, entre ellos enfermeros, psicólogos, terapeutas ocupacionales, trabajadores sociales, técnicos de enfermería, monitores y auxiliares de vivienda.

Se utilizó el muestreo en bola de nieve, en el que un participante indica a otro para acceder a los profesionales^(20)^. Los tres gestores de cada CAPS III, referidos como semillas, al indicar a los primeros participantes que consideraban que tenían experiencias relacionadas con el tema del estudio, favorecieron así la construcción de una red de confianza y credibilidad a lo largo del proceso^(20)^.

El contacto previo, la invitación a participar y la programación de las entrevistas se realizaron tanto en persona, durante las visitas del investigador al servicio, como por mensaje electrónico a través de una aplicación móvil, con el apoyo de los administradores.

Se consideraron los siguientes criterios de inclusión: pertenecer al equipo multiprofesional que atiende a los residentes de edad avanzada de los SRT, tener al menos seis meses de experiencia y no estar de vacaciones ni ausente del trabajo durante el período de recopilación de datos. No participaron en el estudio los profesionales que no prestan cuidados directos a los residentes, como farmacéuticos, técnicos de farmacia, equipo de higiene, servicio de vigilancia y auxiliares administrativos.

### Recolección de Datos

La recolección de datos se llevó a cabo de enero a diciembre de 2024, mediante entrevistas fenomenológicas realizadas por el primer autor, doctorando en ciencias de la salud y enfermero asistencial en ese momento, con amplia experiencia académica en la metodología empleada. Estas entrevistas permiten al participante relatar al entrevistador el significado atribuido al fenómeno vivido y a la acción realizada en su contexto de interacciones sociales^(21)^. Las preguntas desencadenantes fueron: “¿Ha atendido alguna vez a algún residente anciano en el SRT?”, “Cuénteme cómo fue y cuáles fueron las implicaciones de la familia en ese cuidado” y “¿Cuál fue su intención al realizar ese cuidado?”. A partir de ellas, se elaboraron otras preguntas que permitieron al participante profundizar en el tema y compartir sus experiencias. No se realizaron pruebas previas de las preguntas desencadenantes, ya que se definieron con el apoyo del equipo de investigación antes del inicio de la recolección de datos. Al finalizar la entrevista, se recopiló información sobre el tiempo de actuación de los participantes en los SRT y sus experiencias laborales.

Solo el investigador y el entrevistado estuvieron presentes durante las entrevistas, que se realizaron en diversos lugares, como las instalaciones del CAPS III, los SRT y espacios externos, y fueron conducidas por el investigador principal y grabadas en audio, con una duración media de 25 minutos. Hubo una deserción, justificada por motivos personales, momento en el que el investigador volvió a la semilla^(20)^, obteniendo una nueva indicación de participante. No se realizaron entrevistas repetidas. La recolección de datos finalizó cuando el investigador principal comprendió que el fenómeno había sido desvelado, la inquietud había sido resuelta y las ideas en las declaraciones comenzaron a repetirse, habiendo así suficientes estructuras de significado subjetivo para describir lo típico de la acción. En ese momento, no surgió ningún nuevo elemento significativo en los datos, como matices, dimensiones o variabilidad^(17,22,23)^.

### Análisis y Tratamiento de los Datos

Con el fin de garantizar la rigurosidad del proceso de análisis de los datos y entendiendo la fenomenología como un movimiento continuo, en todas las etapas se volvió a los conceptos principales de Schutz (acción social, mundo-vida, trayectoria biográfica, acervo de conocimientos, relación cara a cara, motivos-por qué y motivos-para)^(17,23)^.

Este retorno constante se justifica porque el proceso está mediado por la biografía del investigador, lo que exige el ejercicio mental del distanciamiento para adoptar una actitud científica. Al interpretar y sistematizar las declaraciones de los sujetos, se elabora el tipo vivido, es decir, la acción de objetivar lo subjetivo de los entrevistados^(23)^. Esta elaboración es posible mediante la*epoché*—suspensión de los supuestos del investigador y orientación de la atención hacia sus inquietudes y los objetivos del estudio—y la reducción eidética, que consiste en el análisis consciente elaborado a partir de las unidades de significación de cada individuo^(23)^.

Así, en la fenomenología, el conocimiento se construye a partir de lo vivido en el sentido común, y corresponde al investigador organizar los datos subjetivos, describir la experiencia y llevarla al orden de los significados^(23)^.

A partir del marco teórico de la fenomenología social^(17)^, los datos procedentes de las entrevistas fueron analizados por el investigador principal y validados por el grupo de investigación al que pertenece, sin utilizar*software*, siguiendo los pasos recomendados en estudios basados en el análisis fenomenológico y en los conceptos de Schutz^(17,23)^: transcripción íntegra de cada entrevista, organizando el texto por sujeto a partir de la pregunta orientadora, con el objetivo de aprender el significado individual; lectura y relectura continua de las transcripciones para facilitar la comprensión global de las experiencias de los participantes; recorte de las declaraciones que evidencian las estructuras de los significados subjetivos de la acción, reuniendo los fragmentos que revelan motivos similares; formulación de las categorías de la acción humana en relación con el significado del fenómeno mediante la agrupación de los fragmentos de las declaraciones, distinguiéndolos en “motivos-porque”—que explican la realización de una determinada acción—y “motivos-para”—que corresponden a las intenciones o finalidades que orientan la acción, según se ilustra en la Figura 1.

**Figura 1:** Diagrama resumen del proceso de análisis de datos.

### Aspectos Éticos

Este estudio fue aprobado por el Comité de Ética en Investigación de la Universidad Estatal de Campinas (UNICAMP), bajo el dictamen n.º 4.501.808. Se informó a los participantes de que se trataba de una investigación científica cuyo tema era parte del día a día del investigador principal en su trabajo como enfermero, con el fin de obtener nuevas reflexiones sobre este tema para mejorar la práctica profesional. De conformidad con la Resolución n.º 466/12, los participantes firmaron el Término de Consentimiento Libre y Esclarecido, el Término de Autorización para la Grabación de la Voz y fueron informados de que se trataba de una investigación académica. Con el fin de preservar el anonimato, los relatos se identificaron con la letra “E”, de “entrevistado”, seguida del número correspondiente a la secuencia en la que se realizaron las entrevistas.

## RESULTADOS

Quince participantes formaron parte del estudio, cuatro de ellos técnicos de enfermería, cuatro monitores, dos enfermeros, dos terapeutas ocupacionales, un psicólogo, un asistente social y un auxiliar de vivienda. El tiempo medio de actuación en los SRT fue de cinco años. En cuanto a la experiencia laboral, además de la actuación en salud mental, los participantes también fueron o seguían siendo: cuidadores de personas mayores en atención domiciliaria y residencias de ancianos; vinculados a otras instituciones de salud; o profesionales de sectores no relacionados directamente con la salud, como empleados domésticos, atención al público y educación.

A partir de las entrevistas, fue posible organizar los resultados en dos categorías que constituyen el tipo de experiencia vivida por los participantes en el mundo social a través de las motivaciones, descritas a continuación: “La familia en la experiencia de cuidado del equipo multiprofesional al anciano residente en Servicios Residenciales Terapéuticos”, que caracteriza los “motivos-por qué” del significado intersubjetivo de las experiencias de los participantes en el mundo y las implicaciones familiares en el cuidado de las personas mayores en los SRT desde la perspectiva de los profesionales; y “Intencionalidades del equipo multiprofesional frente a la familia en la experiencia del cuidado”, para describir los “motivos-para” del cuidado realizado por el equipo como acción social, las intencionalidades y lo que esperan de sus acciones a partir de los encuentros cara a cara.

### La Familia en la Experiencia de Cuidado del Equipo Multiprofesional al Adulto Mayor Residente en Servicios Residenciales Terapéuticos

El equipo multiprofesional identifica que los residentes de edad avanzada de los SRT a menudo no cuentan con el apoyo familiar para la prestación de cuidados y que son pocos los casos en los que sería posible reconectar a estos residentes con sus familias, que a veces optan por enviar a los adultos mayores a residencias de ancianos. Denominan estos aspectos como abandono y rechazo, reforzados por la dificultad de establecer contacto con los familiares, que se alejan del cuidado por no poder hacerse cargo de una persona dependiente y debido a historias de conflicto familiar.

*Gran parte de ellos no tienen apoyo familiar. [...] Cuando está en la residencia, tiene todo el apoyo que no tiene en casa. [...] De los residentes que tenemos hoy, que tienen contacto con la familia [...] tres o cuatro, así, estoy hablando de un número muy alto, podrían volver con la familia. – (E6)*

*La mayoría de ellos no tiene familia, y si la tiene, con el tiempo, ya no les cuida, no está cerca. Por eso, me parece bien este proyecto y una residencia terapéutica, ¿sabes? La mayoría, por alguna razón, o por abandono [...] la familia acaba internándolos en una clínica, porque dice que no puede cuidar de ellos, porque antes caminaban, eran independientes, ahora no podemos cuidar de ellos, los internaremos en una clínica. [...] En el caso de la residencia terapéutica, ya son pacientes que no tienen familia. – (E11)*

*Hoy en* día, con *este distanciamiento familiar, he intentado varias veces acercarme a mi hija y ella ni siquiera me responde, no contesta mis llamadas, no quiere saber nada de su madre. [...] Porque muchas veces también está esta historia del anciano con sus familiares, no sabemos cómo fue la vida de este anciano. Hay cuestiones de abandono, de rechazo. Y cuando llega a esa edad, esa persona que ha pasado por todo eso, necesita pasar por todos esos sentimientos y asumirlos. Muchos no pueden, por lo que no quieren contacto. [...] La mayoría de las personas mayores tienen que lidiar con esta cuestión del abandono, y me doy cuenta de que ella intenta mantenerse mucho más al margen, porque la realidad duele mucho. – (E15)*

En el contexto de desinstitucionalización propuesto por la RPB, a menudo el apoyo y los vínculos sociales y familiares de los egresados de camas psiquiátricas están desgastados, y el SRT surge como una alternativa de vivienda para que estas personas no vivan en las calles, expuestas a la violencia y la vulnerabilidad. El servicio se considera una potencialidad que valora la vivienda, donde los residentes son atendidos y apoyados. Sin embargo, para los participantes, el SRT solo debe considerarse cuando se han agotado las inversiones en el núcleo familiar.

*Con todo este proyecto de reforma, cierre de camas y desinstitucionalización, pensamos colectivamente y creamos el proyecto de residencia terapéutica para pacientes que salían de largos períodos de hospitalización, que ya no tenían vínculos sociales, ni familiares, y que ya no iban a vivir dentro de los hospitales y tendrían que vivir en algún lugar. [...] Núcleos familiares muy desgastados, donde las relaciones entre la persona que es el paciente y la red familiar están muy deterioradas [...] indicarle que se inscriba en el programa de residencia terapéutica, o ese paciente acabaría viviendo en la calle o expuesto a la violencia y las vulnerabilidades sociales y su situación de vida empeoraría. [...] Tenemos que invertir todo lo posible en este núcleo*(familiar)*para que este paciente pueda volver a convivir bien y vivir en este núcleo. Si no hay posibilidad, después de agotar todas las inversiones, pensamos en la residencia terapéutica. – (E4)*

*Nunca tuvieron un hogar, nunca tuvieron ese apoyo, ese cuidado, se sienten extraños. [...] Estoy hablando de alguien de más de 60 años, que no tiene apoyo familiar. No sabe lo que es ir a pasear por una plaza, por un parque, porque nunca tuvo esa posibilidad. Creo que esto tiene un enorme potencial. Creo que podemos ofrecerlo como CAPS, como vivienda, yo también como referencia de la casa, valorar este vivir. – (E10)*

*Porque son residentes que no tienen familia, están envejeciendo y están muy bien cuidados, son asistidos, así que creo que no están desamparados. – (E8)*

En algunos casos, los profesionales relatan buenas experiencias con residentes mayores del SRT que aún mantienen vínculos con el núcleo familiar, cuyos familiares visitan la vivienda, lo que contribuye al restablecimiento de la autonomía de la persona. Cuando no es así, los residentes, como parte de la rehabilitación psicosocial, buscan otras formas de familia que no sean consanguíneas, como cuando la comunidad comprende y se integra en el contexto de vida de estos ancianos mediante donaciones y cuando ellos mismos se cuidan y se protegen.

*Tenemos buenas experiencias sobre este proceso de pacientes que hoy en* día *siguen manteniendo el núcleo familiar y la familia va a la casa, los visita, va el* día del *cumpleaños, participa en las felicitaciones, pero* él *vive en la residencia terapéutica y la familia vive allí en su espacio. Esto fue lo que hizo que el paciente, incluso, lograra restablecer un proceso de vida con más autonomía. – (E4)*

*Pienso en la rehabilitación, que abarca desde la cuestión financiera hasta los lazos familiares, que a menudo se rompen, pero nos quedamos con esa cuestión familiar más sanguínea, y ellos crean otras familias, otras redes, que también aportan mucho, y creo que eso forma parte de la rehabilitación. [...] Las fiestas son 100% donaciones. Me parece fantástico, porque son la panadería, el restaurante, el mercado, el bar, las familias los que donan. La vecina que trabaja en la granja dice: “Sé que va a haber una fiesta. Pásate por aquí y llévate dos cartones de huevos”. Eso es apropiarse. Eso es hacer que la comunidad mire y comprenda. – (E15)*

*Cuando salen por la puerta, se cuidan unas a otras. [...] Se dan la mano, se ayudan [...] Aunque en casa se pelean por cualquier cosa, fuera no, fuera se protegen. Creo que, en sus cabecitas, piensan: “Es mi familia. Entonces, voy a cuidar de ellas”, y se cuidan muy bien. – (E2)*

Por último, el equipo multiprofesional, además de considerarse a menudo la familia de los residentes mayores del SRT, al acercar el cuidado que ofrecen a las experiencias personales, también cree que ellos los ven como familiares, ya que colocan al equipo en este lugar, quieren estar cerca todo el tiempo y muestran gratitud, respeto y cariño por los profesionales. Como resultado, al mismo tiempo que sufren por el fallecimiento de los residentes de edad avanzada, ya que pasan más tiempo en el trabajo que en su propia casa, también consideran que el residente desearía tener a alguien realmente de la familia en momentos de fragilidad y enfermedad, porque creen que no es posible ocupar ese lugar como profesionales.

*Mi madre estaba postrada en cama y ya aprendí un poco, así que vine aquí ya sabiendo cómo cuidar a una persona de edad avanzada. – (E1)*

*Muchos tienen familia, otros no tienen a nadie... Su familia, ellos nos ven como su familia, ¿no? Entonces, en el momento en que llegas allí, cuando pasas mucho tiempo sin ir, el* día *que apareces, vienen contándote cosas. [...] Y ves que quieren estar cerca, todo el tiempo. – (E3)*

*Creo que, de todas las escalas, los lugares a los que llamamos “viviendas” son los más importantes. Porque en el entorno los pacientes vienen, permanecen en el entorno y se van a sus casas... En la residencia, no. Lo que tienen como base familiar, por así decirlo, es el personal, los monitores, los auxiliares de vivienda, somos nosotros los que vamos allí a medicarlos. Creo que su familia somos nosotros. No tienen grandes tensiones. Aunque algunos todavía tienen contacto con la familia, una persona lejana, pero creo que es eso. – (E6)*

*Eso me hace seguir siempre en la salud mental, esa gratitud que nunca recibieron. Esa familia que no tienen, intentamos proporcionársela, pero tenemos que tener esa mirada hacia el anciano que va mucho más allá. – (E10)*

*Nos miran con cariño. Nos tienen mucho respeto. Hay algunas que dicen: “Ay, me acuerdo de mi madre. Ella me cuidaba así, ¿verdad?”. [...] Y yo también me siento muy triste cuando fallece. [...] Es como si hubiéramos perdido a alguien de nuestra familia. Porque estamos más aquí que en nuestra casa. [...] Nos ven como si fuéramos familia. [...] Ahora ellos, creo que no piensan así. En esos momentos difíciles... La familia, alguien a su lado, no tienen a nadie allí. Ahí es cuando nos afecta. [...] – (E7)*

*Tampoco podemos fantasear e intentar ocupar otros lugares en su historia, que creo que también tiene que ver con eso, creo que la salud mental tiene eso, esa sensibilización, ese reacercamiento, de la vida y con la familia y todo lo demás, pero también es parte de su historia. – (E5)*

### Intenciones del Equipo Multiprofesional ante la Familia en la Experiencia de Cuidado

Partiendo de la idea de considerar a los residentes de edad avanzada como miembros de la familia, el equipo tiene la intención de realizar el cuidado de la mejor manera, con cariño y de la forma en que les gustaría que se les cuidara a ellos. En los casos en que tienen una red familiar, se identifica la importancia de que el profesional de referencia coordine el cuidado con la familia, además de los demás servicios de la RAPS en los que el residente recibe seguimiento.

*Es bueno, me gusta y trato de cuidar con el mayor cariño, como si fuera uno de los míos, de mi familia. Entonces trato de hacer lo mejor, como quiero que me traten, trato de tratar al prójimo. – (E12)*

*Las discusiones sobre las viviendas deben realizarse dentro del CAPS. El referente debe estar junto con el enfermero del miniequipo del CAPS, junto con el médico. La referencia debe coordinar estos cuidados con la red. Entonces, si es con la UNICAMP, con Mario Gatti, con el centro de salud, con la familia, porque algunos tienen familia y otros no. Y nosotros podemos acompañarlos en estos* últimos *momentos de vida. – (E14)*

El equipo desearía que los residentes de edad avanzada tuvieran familia, para que pudieran estar juntos y tener a alguien a quien visitar, a pesar de las dificultades de convivir con ella. A partir de ahí, identifican que los profesionales del SRT son la familia de estos residentes.

*Me gustaría que todos tuvieran familia. Creo que, por muy difícil que sea vivir con la familia, no hay nada igual, ningún sentimiento igual. Entonces, creo que lo primero era que tuvieran a alguien que los cuidara, ¿sabes? La familia, que pudieran estar juntos, ir a la casa, ir todos. Salir, poder ir... Fui a ver a mi mamá, fui a ver a mi tía, fui a ver a alguien... Fui a la casa de alguien, de un familiar. – (E3)*

*La familia no los acompaña. Nosotros acabamos siendo la familia para ellos. Eso es lo que me hace seguir adelante. – (E13)*

Uno de los miembros del equipo relató una experiencia singular durante su formación, cuando conoció los servicios de vivienda en otro país, un modelo en el que las casas son distribuidas por el municipio y el equipo acude a la residencia en función de la demanda de los residentes, vislumbrando este funcionamiento para los SRT en Brasil.

*Tuve la experiencia de ir a Portugal y conocer Housing First, por ejemplo, que es otra lógica. Sabemos que estamos hablando de otro país, otra cultura, otra política pública. Por lo tanto, no podemos comparar, copiar y pegar el proyecto aquí. [...] Quizás pueda vislumbrar tener esa experiencia aquí. Allí son casas individuales, o un individuo o familias tienen sus propias casas distribuidas por el territorio del municipio y lo hacían a través de visitas o a través de la demanda. [...] Entonces, para limpiar la casa, hacer la compra, organizar las finanzas, a veces, algún seguimiento con un servicio de salud. Entonces, había profesionales que eran referencias de las casas repartidas. Yo, como referente, tendría cinco casas a mi cargo. Pero tendría toda la semana para dedicarme a ese cuidado. – (E9)*

## DISCUSIÓN

El acercamiento a la experiencia vivida por los profesionales que componen el equipo multiprofesional permitió comprender algunos aspectos relacionados con las implicaciones de la familia en la experiencia del cuidado de los adultos mayores residentes en SRT. Las acciones sociales de los participantes revelaron las motivaciones existentes en su mundo-vida, en el que las relaciones con los residentes de edad avanzada en el día a día del cuidado en la RTS construyen el espacio intersubjetivo que permite agregar conocimiento y allanar la trayectoria biográfica^(17)^.

Uno de los principales hallazgos de este estudio se refiere a la inexistencia o fragilidad del apoyo familiar para los residentes de edad avanzada de las SRT. La composición familiar es particularmente importante, ya que la familia se considera la unidad social más básica en la vida humana y parte del mundo vital de las personas, íntimamente relacionada con la prestación de apoyo físico, psicológico y espiritual, además de ofrecer cuidados diarios. En la población de edad avanzada son comunes los sentimientos de soledad y abandono, cuestiones complejas con profundas implicaciones en el bienestar y la salud mental, que requieren enfoques integrados y articulaciones intersectoriales que tengan en cuenta aspectos individuales, relacionales y contextuales^(24)^.

Cabe destacar que las personas de edad avanzada son vulnerables en los ámbitos individual, social y programático^(8)^. Las personas que salen de largas hospitalizaciones psiquiátricas son más propensas a la pérdida de vínculos familiares o a la fragilidad de estos^(10)^. Esta realidad, asociada a las consecuencias del modelo manicomial, constituye uno de los criterios para ingresar en los SRT^(11)^.

Así, la fragilidad o la inexistencia de apoyo familiar entre los residentes de edad avanzada de estos dispositivos revela una ruptura significativa en su mundo-vida. En este sentido, vivir en un SRT no solo significa tener cuidados y vivienda, sino que representa una respuesta de las políticas públicas ante la vulnerabilidad programática de las personas de edad avanzada^(8)^, además de permitir la reconstrucción de redes intersubjetivas y la resignificación de trayectorias^(17)^.

El cuidado familiar está inserido en el mundo-vida y las personas que lo reciben tienen un mejor pronóstico de salud y calidad de vida^(6)^. Sin embargo, la carga y la responsabilidad atribuidas a los familiares también se asocian con altos niveles de sufrimiento psíquico^(6)^. El cuidado de un anciano con psicosis, por ejemplo, en el que las comorbilidades orgánicas son comunes, desafía el acervo de conocimientos disponibles, exige más y es más estresante, ya que rompe con las tipificaciones asociadas al envejecimiento considerado típico^(6)^. Se señala que el familiar que cuida a un anciano con sufrimiento psíquico experimenta desgaste emocional, dificultad para convivir con los síntomas conductuales e identifica la necesidad de compartir la responsabilidad del cuidado con los servicios de salud en un intento por aliviar la sobrecarga^(25)^. Por lo tanto, la enfermedad psíquica de los cuidadores debido a la sobrecarga del cuidado puede incluso afectar su capacidad de cuidar, su calidad de vida y su autoestima, y hacer que tengan comportamientos hostiles, en un círculo vicioso que puede desestabilizar el tejido relacional que sustenta el mundo social, generar conflictos familiares y afectar negativamente la salud de la persona cuidada^(6)^.

Aunque es deber de la familia cuidar a las personas mayores, los familiares no siempre tienen los conocimientos necesarios para proporcionar los cuidados requeridos durante el proceso de envejecimiento, con sus cambios físicos y psicosociales en el mundo-vida^(26)^. Esta situación se intensifica en la sociedad actual, en la que las familias son cada vez menos numerosas, el ritmo de trabajo es más intenso y el cuidado recae, casi siempre, en un miembro de la familia^(26)^. En este contexto, al tensar la tipificación tradicional del papel familiar, es común delegar el cuidado a instituciones de larga estancia, por ejemplo, siendo estas nuevas redes intersubjetivas a menudo consideradas por los familiares como un entorno más adecuado para satisfacer las necesidades de salud de las personas mayores^(26)^. Sin embargo, las personas mayores con sufrimiento psíquico grave presentan una elevada vulnerabilidad y necesidades específicas de cuidados prolongados y, a menudo, se enfrentan a importantes barreras para su admisión en instituciones de larga estancia, que, en general, tienen dificultades para ofrecer apoyo especializado en salud mental, lo que compromete la calidad de los cuidados^(27)^, lo que refuerza el fenómeno de la transinstitucionalización^(7)^y pone de manifiesto un importante reto aún sin superar^(27)^.

Para las personas mayores con trastornos psíquicos y vínculos familiares y sociales frágiles, el SRT se presenta como una alternativa de espacio que valora la vivienda, al proporcionar un entorno intersubjetivo para el desarrollo de relaciones sociales y afectivas para aquellos que hasta entonces se encontraban confinados en hospitales psiquiátricos, teniendo como objetivo principal el rescate de la autonomía y el derecho a vivir en comunidad a través de la rehabilitación psicosocial. Así, las personas que antes eran consideradas pacientes, en el SRT son residentes^(2,28)^y, a partir de su acervo de conocimientos, pueden reconstruir nuevos caminos en su mundo-vida^(17)^. La atención en régimen de manicomio tenía como marcas biográficas las limitaciones de espacio e individualidad, transformadas para el residente del SRT en posibilidades de circular por la comunidad, convivir e interactuar con otras personas a través de relaciones cara a cara, intercambiar experiencias y, sobre todo, aprender del otro y del mundo social que le rodea^(17,28)^. En este proceso, la acción del equipo multiprofesional es fundamental para estimular la vida en el territorio de los residentes de los SRT, el ejercicio de la ciudadanía y la configuración de este espacio como un hogar y no como un lugar de tratamiento^(29)^.

Además, el sentimiento de pertenecer y habitar un espacio tiene un gran significado para las personas que sufren psíquicamente, ya que el hecho de vivir constituye en el mundo-vida una construcción intersubjetiva cargada de significados^(17)^y el “hogar” es, comúnmente, un lugar de acogida, confort, intimidad, intercambios y experiencias de relaciones afectivas e interpersonales a partir del acervo de conocimientos, legitimadas a través de la vida cotidiana doméstica entre los residentes de edad avanzada de la SRT^(28)^. El espacio de vivienda debe adaptarse a las intencionalidades de sus residentes y proporcionar un apoyo flexible por parte de los profesionales, más allá de los patrones externos de organización, ya que “estar en casa” representa la oportunidad de expresar sus subjetividades y el ejercicio de la vida en libertad^(13)^. A partir de ahí, se construye una nueva comprensión del “vivir”: ahora una construcción subjetiva a partir de la biografía de cada residente, que tiene un lugar al que llamar “suyo” en el mundo-vida^(2,13,28)^.

En algunos casos, todavía se identifica la existencia de vínculos entre el residente anciano y sus familiares, que visitan el SRT. El modelo manicomial produce exclusión y distanciamiento del mundo social, con impacto en las relaciones familiares desde las primeras internaciones psiquiátricas, ya que estas personas no suelen recibir visitas durante el tiempo que permanecen en las instituciones, lo que perjudica las relaciones sociales y afectivas^(13)^. La interacción frecuente entre la familia y el anciano a través de visitas al SRT, en los casos en que no viven juntos, no satisface las necesidades de comunicación interpersonal del residente. Sin embargo, este acercamiento favorece la salud física y mental, actualiza los conocimientos y refuerza el sentimiento de pertenencia y continuidad biográfica en el mundo-vida, además de permitir una percepción más positiva del proceso de envejecimiento, lo que repercute en la calidad de vida^(24)^. Según señalaron los participantes, estas visitas se caracterizan por ser encuentros cara a cara y tienen un impacto positivo en contraposición a la exclusión típica de las prácticas manicomiales, lo que puede ayudar a motivar las acciones futuras del residente y del equipo multiprofesional^(17)^.

Con el fin de llenar el vacío de las relaciones familiares frágiles o inexistentes, los residentes de edad avanzada buscan otras formas de familia, ya sea dentro o fuera del SRT, en un movimiento de ocupación del territorio, reconstrucción de la pertenencia al mundo-vida y actualización del acervo de conocimientos^(17)^. Al vivir en la misma residencia, los adultos mayores desarrollan un sentido de pertenencia y de vivir en comunidad, redefiniendo las tipificaciones familiares y ampliando las relaciones afectivas, intersubjetivas y sociales^(17,28)^. En el contexto de la rehabilitación psicosocial, la acción de vivir también se convierte en una forma de apropiación material y simbólica de los espacios de la casa, donde la convivencia se consolida como un proceso de intercambio de recursos, cuidados y afectos entre los residentes^(28)^. Sin embargo, existe una centralidad de los SRT en la red de servicios de salud, lo que puede restringir la reinserción social de los residentes y dificultar la creación de nuevos vínculos^(13)^. Este hecho apunta a la necesidad de pensar las acciones de cuidado desde una perspectiva intersectorial^(3,8,13)^.

Además, la superación del modelo manicomial exige la participación de los residentes de edad avanzada en la ciudad, teniendo en cuenta la porosidad del tejido urbano proporcionada por los SRT, mediante la inserción en grupos sociales y/o espacios públicos y comunitarios, lo que promueve la ampliación del mundo-vida y la resignificación de las tipificaciones sociales asociadas a la exclusión^(17,28)^. Esta forma de participación social fortalece la intersubjetividad y puede transformar la vida cotidiana, el recorrido biográfico y las condiciones de vida de las personas mayores, que a menudo están marcadas por la enfermedad, la violencia, la injusticia social, la desigualdad, los prejuicios, la exclusión y la opresión^(28)^. En la reinserción social, las actitudes de la comunidad son fundamentales, sobre todo cuando se busca promover la concienciación sobre la salud mental a través del contacto directo con los residentes de los SRT, acciones que ayudan a reducir el estigma y a fortalecer el modelo de atención en libertad^(30)^. Como consecuencia de vivir en sociedad, los residentes amplían su comunicación interpersonal, cultivan pasatiempos y mejoran su calidad cultural al valorar la subjetividad y el potencial de los encuentros^(28)^.

En este contexto, los propios profesionales se consideran familia de los residentes de edad avanzada. La autonomía en el SRT, en comparación con las instituciones de tipo asilar, es superior debido principalmente a la construcción de la interdependencia entre los dispositivos, el territorio y las personas, considerándose en este último caso también la relación cara a cara entre residentes y profesionales para producir nuevos significados^(28)^. La construcción de estas relaciones es parte integrante del proceso de desinstitucionalización, ya que la posibilidad de tomar decisiones permite la reconstrucción biográfica y la resignificación de los significados y los acervos de conocimiento de su mundo-vida^(17,28)^. Además, las acciones de los profesionales en la vida cotidiana van mucho más allá de las cuestiones físicas y psicosociales. Se trata de una perspectiva de cuidado relacional y terapéutico, expresada a través de la escucha cualificada, la comunicación interpersonal y la construcción de vínculos con nuevos significados en el mundo-vida, que también proporcionan consuelo emocional y espiritual^(17,31)^. Esta interacción revela el potencial del enfoque multiprofesional en salud mental mediante el cuidado centrado en la persona y la valoración de la subjetividad^(31)^, aspectos fundamentales para la rehabilitación psicosocial.

Sin embargo, es necesario que los profesionales tengan cuidado al considerar al residente anciano como un familiar, ya que esto demuestra una ambigüedad en sus funciones sociales y terapéuticas, necesarias para su actuación en el SRT^(2,10)^. Esta actitud de cuidado se asocia con la caridad, restringe la intersubjetividad y reproduce la idea de aislamiento social perpetuada históricamente en los manicomios, ya que es necesario redefinir el cuidado de manera que se considere que estos dispositivos necesitan intersectorialidad y conocimientos técnicos, alineándolos con las motivaciones orientadas al fortalecimiento y la construcción del mundo-vida de los residentes de edad avanzada^(2,10,17)^. Aunque existen, las acciones intersectoriales en el ámbito de la atención en la RAPS se llevan a cabo a través de atenciones compartidas y reuniones, a menudo de manera restringida y aislada, sin una sistematización efectiva^(3)^. Este contexto refleja una red fragmentada y centralizada en los servicios especializados, pero que necesita articularse de forma fortalecida entre niveles y servicios para garantizar una atención longitudinal, territorial y basada en el conocimiento técnico^(3)^. Así, la comprensión fenomenológica contribuye al revelar que la acción de cuidar del equipo multiprofesional está impregnada de una actitud caritativa, característica asociada a la experiencia previa de las personas y con un fuerte atractivo social, lo que puede distanciarse de las prácticas basadas en el conocimiento técnico y científico^(17)^.

Así, cabe destacar que el modelo actual de atención de salud mental aún enfrenta obstáculos en la efectividad de la atención en libertad en el territorio^(3,13,29)^. Persisten las propuestas que fomentan la hospitalización psiquiátrica y la financiación de comunidades terapéuticas basadas en una visión prohibicionista y excluyente de la persona que sufre psíquicamente^(16)^. Estas acciones reflejan intentos de retomar el modelo manicomial, lo que refuerza la importancia de reflexiones críticas sobre las prácticas en los servicios de salud^(13,16)^. En este contexto, destaca la acción de los profesionales, cuya actuación debe estar alineada con los principios de la rehabilitación psicosocial y la garantía de derechos, con el fin de superar las lógicas institucionales aún vigentes^(13)^.

Los profesionales informan que atienden a los residentes de edad avanzada del SRT de la forma en que les gustaría que los atendieran a ellos, lo que demuestra empatía. La empatía es un constructo multidimensional que incluye factores cognitivos y afectivos, y se considera como la comprensión, por parte del profesional, de los sentimientos del residente, aspecto esencial en contextos de salud mental, en los que las personas que sufren psíquicamente valoran la perspectiva de los profesionales y su comprensión de la situación^(32)^. La empatía ejercida por los profesionales del SRT surge del mundo-vida compartido y de las relaciones cara a cara, lo que permite comprender las experiencias de los residentes a partir de sus conocimientos y de las tipificaciones de lo vivido^(17)^. La capacidad empática también permite adoptar posiciones no defensivas y facilita la obtención de resultados satisfactorios y productivos^(32)^. Sin embargo, en el contexto de la atención de salud mental, la empatía no puede verse solo como una característica personal, sino como una competencia relacional que se convierte en un elemento central de la relación terapéutica^(31)^, lo que permite una escucha cualificada, el reconocimiento de la singularidad y el fortalecimiento de los vínculos con los residentes de los SRT. Así, la empatía puede entenderse como una perspectiva que debe impregnar la actuación de todo el equipo multiprofesional, como un aspecto esencial de la atención^(32)^.

Los participantes identifican la importancia de articular la atención con los familiares y otros servicios en los que el residente de edad avanzada recibe seguimiento, lo que representa un proceso de ampliación de la intersubjetividad y de actualización de los conocimientos sobre las singularidades de los residentes, y demuestra la necesidad de que los profesionales del SRT realicen orientación, educación y apoyo a los familiares y trabajadores de otros contextos de salud^(33)^. Esta práctica puede entenderse como una estrategia para hacer frente a la vulnerabilidad programática de las personas mayores y a la fragmentación de la red de servicios, al permitir que los profesionales coordinen acciones para ampliar el acceso, fortalecer (fortalecer) los vínculos y garantizar la continuidad de la atención sanitaria^(3,8)^. Como consecuencia, puede producirse un acercamiento del residente al contexto familiar y a otros espacios de la comunidad, con el fin de contribuir a la participación social y a la reorganización del mundo vital de las personas mayores, en consonancia con proyectos orientados a la autonomía y la inclusión^(28)^.

Otras observaciones de los profesionales fueron que les gustaría que los residentes de edad avanzada tuvieran familia y, por eso, se ponen en el lugar de los familiares. En cuanto a la empatía, esta es un factor importante para el desarrollo de la relación entre el residente y el profesional, ya que estrecha las relaciones cara a cara y la confianza para el cuidado, y es necesario tener cuidado de no sobrepasar los límites de los roles sociales tipificados, fundamentales para la claridad de las funciones en el SRT^(2,10,32)^.

Como alternativa, en consonancia con la rehabilitación psicosocial, el profesional puede, a partir de los conocimientos de los residentes, ayudar a recuperar, reconocer y validar los deseos de las personas mayores de reencontrarse con amigos, familiares, lugares y relaciones que experimentaron a lo largo de su vida antes de la hospitalización psiquiátrica, con el objetivo de revivir momentos de felicidad y protección^(28)^.

Por último, los profesionales identifican el deseo de que la atención al público del SRT se realice fuera de las instituciones, por ejemplo, mediante visitas a residencias repartidas por el territorio, lo que revela las acciones orientadas a la motivación futura que visa la construcción de prácticas alineadas con el mundo-vida del residente^(17)^. Esta idea se alinea con iniciativas en todo el mundo, como Housing First, que, a diferencia del movimiento de desinstitucionalización evidenciado internacionalmente^(7)^, prioriza la vivienda inmediata e independiente, valora la autonomía y la elección de la persona^(34)^, características que apuntan cada vez más al cuidado fuera de los hospitales y las instituciones comunitarias, realizado en la propia casa de la persona, cuando sea posible^(35)^. Para ello, es necesario que los profesionales de la salud se conviertan en agentes activos en los debates sobre el aislamiento social y las necesidades de salud mental, especialmente de la población de edad avanzada^(35)^. Se revela que las motivaciones futuras de los participantes se encuentran en la intención de cuidar la salud mental en libertad en las distintas etapas de la vida mediante la valoración de la trayectoria biográfica, sacando a la luz problemas como el aislamiento de las personas mayores que sufren psíquicamente^(17)^.

Este estudio pone de manifiesto las prácticas del equipo multiprofesional en el ámbito de las implicaciones de la familia en el cuidado diario de las personas mayores que viven en los SRT, destacando la importancia de esta dimensión en su mundo-vida. El enfoque centrado en las singularidades del público anciano en los dispositivos de rehabilitación psicosocial apunta a la relevancia de ampliar el debate sobre las necesidades intersubjetivas de estas personas y cualificar a los profesionales para el cuidado, entre ellos el equipo de enfermería, teniendo en cuenta el escenario del envejecimiento de la población.

En cuanto a las limitaciones del estudio, cabe destacar que los participantes son profesionales que trabajan en algunos SRT del municipio donde se realizó la investigación, por lo que los resultados están vinculados a las experiencias, percepciones y contextos específicos de los sujetos involucrados^(17)^. Sin embargo, este estudio no se propuso analizar otras características que pueden influir en el mundo vital y la acción cotidiana en los SRT. Se recomienda que las investigaciones futuras exploren otros aspectos estructurales, históricos y sociales, especialmente en relación con la participación y el papel de la familia en el proceso de desinstitucionalización y rehabilitación psicosocial de las personas mayores, ampliando la comprensión sobre los retos y las potencialidades de este cuidado en diferentes realidades socioculturales.

## CONCLUSIÓN

En este estudio, fue posible construir el tipo vivido a partir de las categorías temáticas que evidenciaron las motivaciones de las experiencias de los participantes, estando estas experiencias impregnadas por las implicaciones de la familia en el mundo-vida del residente anciano del SRT, lo que influye en el cuidado realizado por el equipo multiprofesional.

A partir de los resultados, se observa que la trayectoria biográfica de los residentes de edad avanzada del SRT está marcada por la vulnerabilidad, la ausencia, la fragilidad y la ruptura de los vínculos familiares, lo que sitúa a los SRTS como espacios importantes en el proceso de desinstitucionalización y rehabilitación psicosocial. La actuación de los profesionales en el día a día, a través de la escucha cualificada y la empatía, permite reinterpretar las experiencias de exclusión y sufrimiento de los residentes.

En este contexto, se observa que la ruptura de las relaciones en el mundo-vida convierte a los SRT en una alternativa potente de vivienda y cuidado, con la posibilidad de acoger y reconstruir vínculos afectivos, intersubjetivos y sociales, ya sea entre residentes que aún mantienen contacto con sus familias de origen o mediante la formación de nuevos arreglos relacionales dentro de la propia casa o en la comunidad. El equipo multiprofesional también reconoce su función de referencia afectiva y articuladora de las acciones en el mundo-vida de los residentes para garantizar un cuidado calificado y humanizado a partir de la biografía previa y la reconstrucción de significados en la vida cotidiana. A pesar de ello, en momentos de mayor fragilidad emocional, tanto el equipo como los residentes desearían contar con el apoyo de los familiares.

La intencionalidad de las acciones del equipo multiprofesional en los SRT está marcada por la búsqueda de una atención cualificada para los residentes de edad avanzada. Cuando hay presencia de familiares, aunque esté impregnada de conflictos, el equipo espera que el profesional de referencia articule la relación entre ellos, además de la expectativa de que los residentes puedan contar con sus propias familias. Sin embargo, las diferentes formas de organización de estos espacios también influyen en las experiencias, lo que indica que la forma en que se estructura el servicio puede potenciar o limitar la relación con la familia.

Además, los resultados de este estudio apuntan a la necesidad de que la atención multiprofesional en los SRT, principalmente por parte de los profesionales que están más cerca de los residentes, como el equipo de enfermería, se oriente por una acción ampliada y por la articulación intersectorial, reconociendo las singularidades de los residentes con la perspectiva de superar las prácticas fragmentadas en los servicios de la red. Así, estas acciones pueden entenderse como estrategias fundamentales para hacer frente a la vulnerabilidad programática de las personas mayores. Por último, los deseos expresados por los profesionales de contar con modelos de atención territorializados e integrados revelan la motivación de la acción alineada con los principios de la rehabilitación psicosocial, el ejercicio de la ciudadanía y el derecho a existir en la sociedad.

## DISPONIBILIDAD DE DATOS

El conjunto de datos que respaldan las conclusiones de este estudio está disponible previa solicitud al autor correspondiente.
